# Novel adenovirus vaccine vectors lacking thrombosis-associated interactions with platelet factor 4

**DOI:** 10.1016/j.isci.2025.114329

**Published:** 2025-12-04

**Authors:** Erwan Sallard, Daniel Pembaur, Matias Ciancaglini, Lucie Manov-Bouard, Denice Weklak, Firas Hamdan, Chun Kit Chan, Franziska Jönsson, Elise Chabot, Carmen Musielak, Elena Scurti, Sara Feola, Sebastian Schellhorn, Nissai Beaude, Katrin Schröer, Daipayan Sarkar, Georgia Koukou, Xiaoyan Wang, Natascha Schmidt, Wibke Bayer, Malik Aydin, Vera Kemp, Alan L. Parker, Dirk Grimm, Tapani Viitala, Vincenzo Cerullo, Abhishek Singharoy, Alexander T. Baker, Wenli Zhang, Daniel Pinschewer, Florian Kreppel, Anja Ehrhardt

**Affiliations:** 1 Virology and Microbiology, Center for Biomedical Education and Research (ZBAF), Department of Human Medicine, Faculty of Health, Witten/Herdecke University, 58453 Witten, Germany; 2 Chair of Biochemistry and Molecular Medicine, Center for Biomedical Education and Research (ZBAF), Department of Human Medicine, Faculty of Health, Witten/Herdecke University, 58453 Witten, Germany; 3 Division of Experimental Virology, Department of Biomedicine, University of Basel, 4051 Basel, Switzerland; 4 Accession Therapeutics Limited, OX4 2JZ Oxford, UK; 5 University of Helsinki, Faculty of Pharmacy, Laboratory of Immunovirotherapy, Drug Research Program Helsinki, Uusimaa, Helsinki, Finland; 6 Helsinki Institute of Life Science (HiLIFE), Fabianinkatu 33, University of Helsinki, 00710 Helsinki, Finland; 7 Translational Immunology Program (TRIMM), Faculty of Medicine Helsinki University, Helsinki, Finland; 8 Digital Precision Cancer Medicine Flagship (iCAN), University of Helsinki, Helsinki, Finland; 9 Biodesign Institute Center for Applied Structural Discovery & Biosense Network, School of Molecular Science, Arizona State University, Tempe, AZ, USA; 10 NIH P41 Biotechnology Resource Center for Macromolecular Modeling and Bioinformatics, Beckman Institute for Advanced Science and Technology, University of Illinois at Urbana Champaign, Champaign, IL, USA; 11 École Normale Supérieure de Lyon, Lyon 1 Claude Bernard University, Lyon, France; 12 University of Helsinki, Faculty of Pharmacy, Drug Research Program, Division of Pharmaceutical Chemistry and Technology, 00014 Helsinki, Finland; 13 AgroParisTech, Paris-Saclay University, Saclay, France; 14 MSU-DOE Plant Research Laboratory, East Lansing, MI, USA; 15 Institute for Virology, University Hospital Essen, University Duisburg-Essen, Essen, Germany; 16 Laboratory of Experimental Pediatric Pneumology and Allergology, Center for Biomedical Education and Research (ZBAF), Department of Human Medicine, Faculty of Health, Witten/Herdecke University, 58453 Witten, Germany; 17 Department of Cell & Chemical Biology, Leiden University Medical Center, Leiden, the Netherlands; 18 Division of Cancer and Genetics, Cardiff University School of Medicine, Heath Park, CF14 4XN Cardiff, UK; 19 Department of Infectious Diseases/Virology, Section Viral Vector Technologies, Medical Faculty and Faculty of Engineering Sciences, University of Heidelberg, BioQuant, 69120 Heidelberg, Germany; 20 Åbo Akademi University, Faculty of Science and Engineering, Pharmaceutical Sciences Laboratory, 20520 Turku, Finland; 21 Department of Molecular Medicine and Medical Biotechnology and CEINGE, Naples University Federico II, Naples, Italy

**Keywords:** Molecular biology, Immune response

## Abstract

The adenoviral vector-based AstraZeneca and Janssen COVID-19 vaccines have been associated with rare cases of thrombosis, believed to be triggered, among other factors, by vector binding to the blood protein platelet factor 4 (PF4). To identify vectors with lower thrombosis risk, we screened 50 natural and hexon-modified adenoviruses (Ads). Unlike the applied COVID-19 vaccines and most tested vectors, Ad34 and Ad80, as well as Ad5 vectors with deleted or chemically shielded hexon hyper-variable region 1 (HVR1), did not bind to PF4. Furthermore, interactions with PF4 substantially modified Ad5 infectivity in various immortalized and primary cells, suggesting that PF4 may influence existing vector tropism. Finally, HVR1-deleted Ad5 and Ad34 vectors expressing SARS-CoV-2 spike S1 domain were tested as vaccine candidates in mice and induced robust cellular immune responses. Therefore, the identified PF4 non-binding vectors may represent safe and efficient candidates for clinical applications.

## Highlights

• We developed higher-throughput methods to study adenovirus binding to PF4
• Ad5-ΔHVR1, Ad34, and Ad80 do not bind to PF4
• Ad5-ΔHVR1 was non-inferior to Ad5 as a vaccine vector in mice
• PF4 modified the infectivity of adenoviruses that bind PF4

## Graphical abstract

**Figure:**
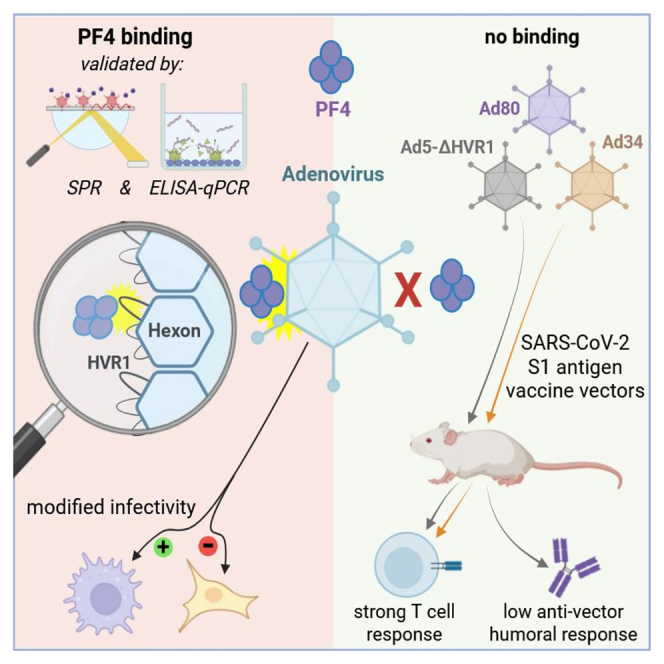

## Introduction

Adenoviruses (Ads) are non-enveloped viruses with a linear double-stranded DNA genome comprising between 26 and 48 kb.^1^^,^^2^ There are currently 116 known Ad types infecting humans,^3^ classified into seven species, and an even larger diversity of non-human Ads infecting other species, including primates. Adeno-associated viruses (AAVs) are parvoviruses with an approximately 5 kb long single-stranded DNA genome, and in nature depend on adenovirus or herpesvirus coinfections to replicate.^4^ Due to their high manufacturability, gene delivery efficiency, and genetic stability, recombinant AAVs and Ads are the most prominent type of viral vectors used in gene therapy and vaccine development,^5^ respectively. Notably, the Vaxzevria (ChAdOx1-nCoV19, derived from chimpanzee Ad type Y25, from AstraZeneca) and Jcovden (Ad26.COV2.S, derived from human Ad26, from Janssen) COVID-19 vaccines have already been administered ≥2 billion times,^6^ and established Ad vaccines as one of the most powerful tools against pandemics. Despite the concomitant success of mRNA vaccines, Ad COVID-19 vaccines remained critical in regions with unstable cold-chain logistics^7^ and tended to induce stronger T cell immunity.^8^^,^^9^

However, clinical applications of Ad vectors still face several obstacles, among which are the ability of certain Ad types to interact with blood proteins after systemic administration or local injection, including with prothrombin, the most abundant coagulation factor.^10^ Moreover, Ad type 5 (Ad5) displays a strong and potentially pathological liver tropism due to its binding to the coagulation factor X^11^^,^^12^ on the fifth and seventh hypervariable regions (HVRs) of its hexon protein,^13^ the most abundant Ad capsid protein. Likewise, the ChAdY25 and Ad26 vaccines have been associated with very rare cases of vaccine-induced immune thrombotic thrombocytopenia (VITT, also termed TTS), with an incidence in the order of magnitude of 1 case per 100,000 vaccinated persons.^14^ Patients present with various symptoms within 5–20 days after the first vaccine injection, including cerebral venous sinus thrombosis, splanchnic vein thrombosis, or other unusual thromboembolic events.^15^ VITT was fatal in 23–40% of cases^16^^,^^17^ in the months following its identification, but fatality rates have been drastically reduced by increased physician awareness and updated intervention guidelines. VITT is thought to be initiated by the binding of the vectors to platelet factor 4^15^^,^^18^ (PF4, also known as CXCL4), which could activate in a small subset of patients a cascade of immune reactions, notably the production of auto-antibodies, and lead to severe adverse effects. PF4 is a 7.8 kDa cationic protein secreted by activated platelets, whose physiological functions include the recruitment of platelets to glycosaminoglycans exposed in vascular injuries and the opsonization of the negatively charged surfaces of pathogens.^19^ PF4 blood concentration is usually around 10 ng/mL, but can reach 3–15 μg/mL in case of platelet activation.^20^^,^^21^ Certain AAV vectors have also been associated with a thrombotic disorder termed thrombotic microangiopathy (TMA) that occurred in several patients enrolled in high dose AAV gene therapy trials, although no link with PF4 has been established to date.^22^^,^^23^

The development of safer vaccine and gene therapy vector platforms may protect patients from rare but fatal side effects and improve public trust in medical treatments and prophylaxes. Therefore, we aimed to characterize PF4 interactions with viral vectors, which, contrary to VITT late stage mechanisms following auto-antibodies secretion, were still relatively poorly understood, and to identify Ad and AAV types with low or absent PF4 binding. Here, we screened a collection of 44 natural human and simian Ad types, 6 hexon-modified Ad5 variants, and 12 AAV serotypes using surface plasmon resonance (SPR) and the novel ELISA-qPCR and Aggregate Pull-Down techniques. We found that Ad34, Ad80, and Ad5 variants with HVR1 deletion or shielding lacked PF4 binding. This confirmed the hypothesis that PF4 binds adenoviruses on the hexon protein, and identified the HVR1 hexon loop as one critical interaction site. Moreover, we found that PF4 substantially modified Ad5 attachment and infection levels in numerous immortalized or primary cell types. Finally, we showed that PF4-negative vectors can be used as vaccine vectors *in vivo*, and in particular that an HVR1-deleted Ad5 vaccine vector displayed a promising immunogenicity profile.

## Results

### Screenings of adeno-associated viruses and adenoviruses collections identify vectors lacking PF4 binding

**Figure 1:**
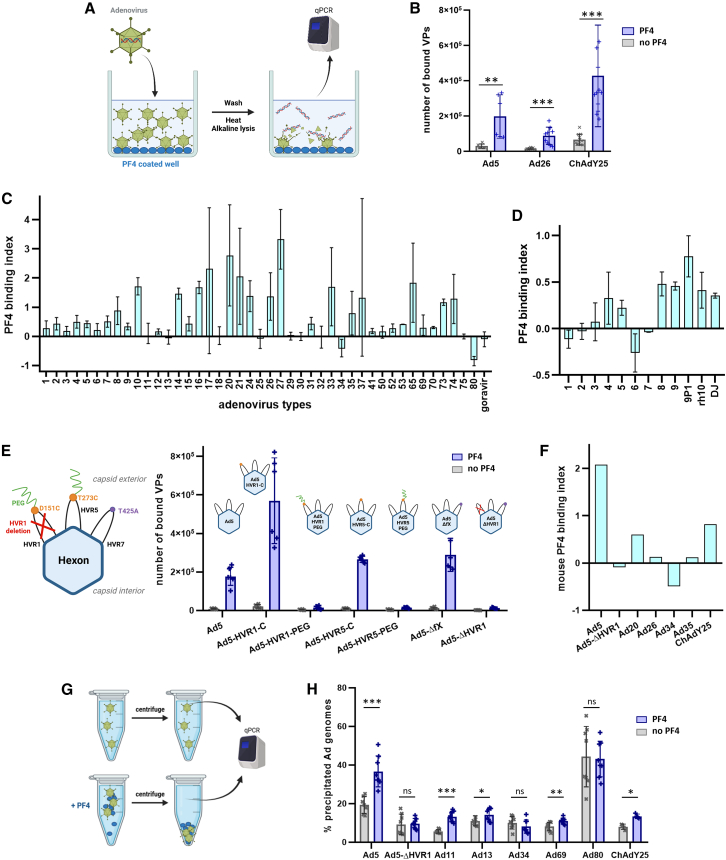
Identification of adenovirus and adeno-associated virus vectors lacking binding to PF4 by ELISA-qPCR and aggregate pull-down screening (A) Principle of the ELISA-qPCR technique. Adenovirus (Ad) vector particles (VPs) are allowed to interact with proteins, e.g., PF4, coated on an ELISA plate. After washing, the genomes of VPs that specifically interact with the proteins are released by heating and alkaline treatment and quantified by qPCR. Figure created with BioRender. (B) PF4 binding of vaccine-equivalent vectors. Ad5 was obtained from the Ad-GLN collection. *N* ≥ 6, two independent repeats. Data are represented as mean ± standard deviation. Two-sided Mann-Whitney U tests. The significance threshold was set at *p* < 0.05. Significance symbols: ns = non-significant, ∗ = *p* < 0.05, ∗∗ = *p* < 0.01, ∗∗∗ = *p* < 0.001. (C and D) Screening of human and gorilla Ad (C) and AAV (D) collections for PF4 binding by ELISA-qPCR. For each experiment repeat, the PF4 binding index is computed as described in STAR Methods (Statistics), with positive values indicating significant binding to PF4 and negative values corresponding to overlap in the number of bound VPs in PF4-coated versus control samples. Averages and minimum/maximum range of the PF4 binding index from two to four (Ad5, Ad11, Ad34, and Ad80) independent repeats are displayed. Data are represented as mean ± standard deviation. (E) ELISA-qPCR of Ad5 hexon genetic and chemical variants for PF4 binding. Studied variants include: D151C and T273C point mutations; a 5 kDa polyethylene-glycol (PEG) polymer covalently linked to a cysteine residue, which prevents binding on part of the hexon surface by steric competition; deletion of the HVR1 loop; and the T425A substitution known to ablate the binding of factor X.^12^ The E1-deleted, GFP-expressing Ad5 vector was used as control (Ad5). HVR: hyper-variable region. PEG: polyethylene glycol. Schematics created with BioRender. *N* = 6, two independent repeats. Data are represented as mean ± standard deviation. (F) ELISA-qPCR of selected Ads for binding to mouse PF4. The binding index is computed as described in STAR Methods (Statistics). Measurements were performed once with technical triplicates. Data are represented as mean. (G) Principle of the aggregate pull-down technique. Aggregates forming upon interaction with PF4 are separated from free VPs by low speed centrifugation and titrated by qPCR. Figure created with BioRender. (H) Aggregate pull-down of selected Ads in the absence or presence of PF4. *N* = 8, two independent repeats. The aggregation rate was calculated based on the titration of Ad genomes both in the pellet (aggregates) and in the supernatant (free VPs). Data are represented as mean ± standard deviation. Two-sided Mann-Whitney U tests. The significance threshold was set at *p* < 0.05. Significance symbols: ns = non-significant, ∗ = *p* < 0.05, ∗∗ = *p* < 0.01, ∗∗∗ = *p* < 0.001.

In order to rapidly screen large vector collections, we established the ELISA-qPCR technique (Figures 1A and S1). Briefly, virus particles are incubated with proteins of interest coated on a microtiter plate; following washes, vectors that bound to the proteins remain in the wells and can be quantified by qPCR. We confirmed the specificity of ELISA-qPCR in the case of a few known interactors of Ad5 (Figures S1A and S1B), observed equivalent recovery rates between Ad5 variants (Figure S1C), and were able to replicate previous results^18^ by detecting PF4 binding to Ad5, ChAdY25, and Ad26 (Figure 1B) as well as the calcium-dependency of coagulation factor X binding to Ad5^24^ (Figure S1B). We then screened for PF4 binding a collection of 43 natural human Ad types drawn from all known human Ad species^25^ as well as goravir, a species B oncolytic vector with the capsid of a gorilla Ad.^26^ We found that Ad34 and Ad80 were the only tested types for which PF4 binding could not be detected in any of the experimental repeats, as indicated by a consistently negative binding index (Figure 1C). Likewise, a screening of 12 AAV serotypes recapitulated the previous finding^27^ that AAV8 and 9, but not 1 and 6, bound to PF4 (Figure 1D). Among the other tested AAV vectors, only AAV7 did not display detectable PF4 binding using the same criteria.

Baker et al. predicted by Brownian Dynamics modeling that PF4 binds to Ad hexon HVRs, with HVR1 being the most likely binding site for the ChAdY25 vaccine.^18^ To gather further information on the location of PF4 binding site(s), we compared by ELISA-qPCR the PF4 binding of Ad5 variants with chemically or genetically modified hexons (Figure 1E). Point mutations in HVR1, HVR5, and HVR7 did not prevent PF4 binding, whereas the deletion of the full HVR1 loop abolished PF4 binding (Ad5-ΔHVR1). PEGylation of HVR1 and HVR5, i.e., covalent linking of a large inert polymer which sterically prevents interactors from binding near its linkage site, also inhibited PF4 binding (Figure 1E). ELISA-qPCR screening of a select adenovirus panel for binding to mouse PF4 yielded the same results as human PF4, namely that Ad5-ΔHVR1 and Ad34 did not bind to mouse PF4, whereas Ad5, Ad20, Ad35, and the vaccine-equivalents Ad26 and ChAdY25 did (Figure 1F).

To validate these observations, we established the aggregate pull-down (APD) technique to quantify Ad vector particle (VP) aggregates that may form upon interaction with human PF4 and be segregated from free VPs by low speed centrifugation (Figure 1G). As expected, we observed PF4-induced aggregation of Ad5, Ad11, Ad13, Ad69 and ChAdY25, but not of Ad34, Ad80, and Ad5-ΔHVR1 (Figure 1H).

**Figure 2:**
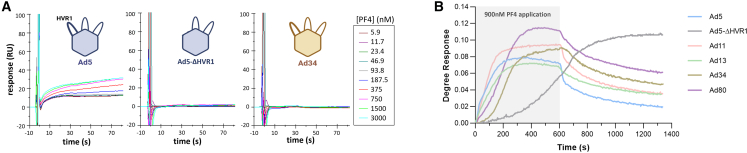
Surface plasmon resonance identifies different PF4 binding patterns among adenoviruses (A) Surface plasmon resonance (SPR) measurements of PF4 binding to Ad5, Ad34, and Ad5-ΔHVR1. Measurements were conducted in PBS +0.5% BSA +0.005% P20 over 90 s injection time with varying PF4 concentrations. (B) SPR measurements of PF4 binding to indicated Ads were conducted in pure PBS with 600 s injection (grey-colored phase) using a PF4 concentration of 900 nM = 7 μg/mL, followed by 750 s flush. Representative traces are displayed.

Finally, the surface plasmon resonance (SPR) technique confirmed that Ad34 and Ad5-ΔHVR1 lacked detectable PF4 binding (Figure 2A). To gain mechanistic insights into PF4 binding to Ads, we conducted additional SPR measurements with longer injection times and in the absence of passivating agents to decrease the detection threshold. Ad5, Ad11, and Ad13 interacted with PF4 with relatively fast kinetics of association and dissociation (Figures 2B and S2B). A curve model fit suggested the coexistence of two interaction patterns (Figure S2A) with a likely electrostatic component followed by a slower dissociating, likely hydrophobic interaction. On the contrary, Ad80, Ad34, and especially Ad5-ΔHVR1 displayed a cooperative binding with slow or absent dissociation. The lack of passivating agents in this experiment and the very slow association kinetics of this interaction may explain why it was not observed in other assays.

### Adenoviruses type 5 binding to platelet factor 4 is dependent on its hexon hyper-variable region 1 loop

**Figure 3:**
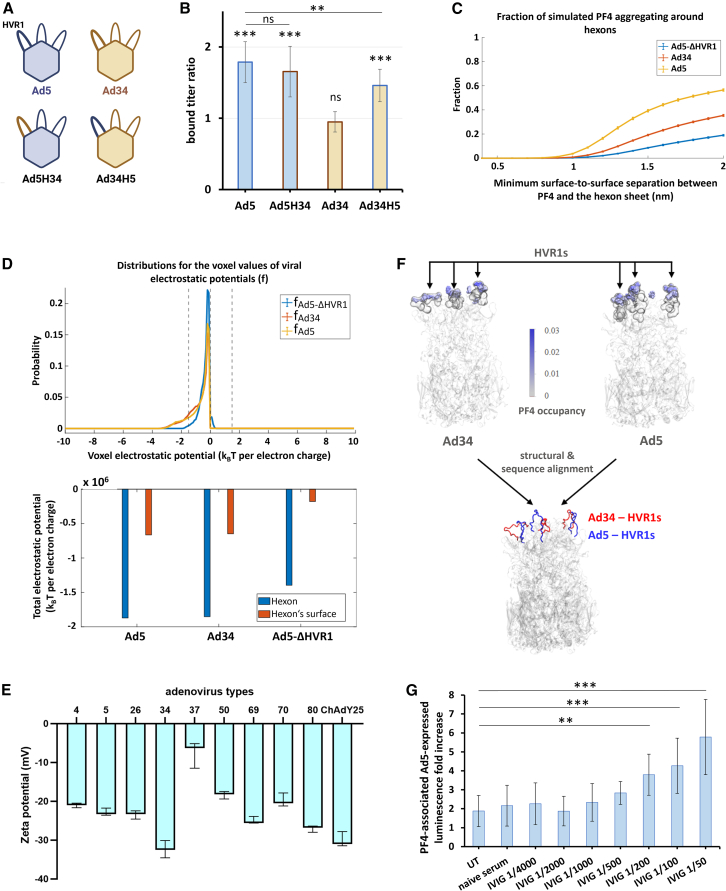
PF4 likely binds to Ad5 hexon HVR1 loop and partially protects Ad5 against neutralizing antibodies (A) Schematic representation of the HVR1 exchange performed to construct the Ad5H34 and Ad34H5 vectors. Figure created with BioRender. (B) ELISA-qPCR of the Ad5H34 and Ad34H5 variants for PF4 binding. Numbers of bound VPs in PF4 coated wells were normalized on the average number from the non-coated wells of the same experiment repeat. *N* = 8, three independent repeats. Significance levels on top of bars represent the comparison (Mann-Whitney U test) of bound titers with versus without PF4. Pairwise comparisons were also conducted between bound titer ratios of Ad5 versus Ad5H34, and of Ad5 versus Ad34H5. Data are represented as mean ± standard deviation. The significance threshold was set at *p* < 0.05. Significance symbols: ns = non-significant, ∗ = *p* < 0.05, ∗∗ = *p* < 0.01, ∗∗∗ = *p* < 0.001. (C) Fraction of PF4 found at given surface-to-surface distance from adenovirus hexons, as sampled from Brownian dynamics (BD) simulations. (D) Distribution of computed surface electrostatic potential as well as integrated values across the whole hexons or hexon surface. (E) Surface potential of Ad particles measured by electrophoretic light scattering (ELS). Data are represented as mean ± standard deviation. *N* = 3. (F) BD simulations of popular regions for PF4 occupancy on hexons. Structural alignments of Ad34’s hexon and Ad5’s hexon suggest that Ad5’s HVR1 loops protrude more than those of Ad34, potentially enhancing their likelihood to interact with PF4 in the bulk solvent. Detailed molecular images mapping popular PF4 interacting residues to their molecular positions in either Ad34’s hexon or Ad5’s hexon are given in Figure S3B. (G) PF4 interference assay with Ad5 human serum neutralizing antibodies. Luciferase-expressing Ad5 vectors from the GLN collection were incubated with or without 10 μg/mL PF4 and 1/50 diluted human seronegative serum (“naive serum”) or pooled human intravenous immunoglobulins (“IVIG”) at varying dilutions. A549 cells were then infected with the suspensions at 500 VP/cell (vpc), and luciferase luminescence was measured at 24 hpi. The ratio of luminescence levels between samples with and without PF4 that received identical serum or immunoglobulin treatment is displayed. UT: untreated, without human serum or antibodies. *N* = 12, four independent repeats. ANOVA test of displayed results yielded *p* < 0.0001, and Dunnett post-hoc tests were conducted against the “UT” sample for all other samples. Pairwise comparisons that did not yield significant *p*-values are not displayed on the figure. Data are represented as mean ± standard deviation. The significance threshold was set at *p* < 0.05. Significance symbols: ∗ = *p* < 0.05, ∗∗ = *p* < 0.01, ∗∗∗ = *p* < 0.001.

Based on SPR results, we hypothesized that the positively charged PF4 protein is recruited on Ad5 capsid by the negatively charged HVR1 loop, but that electrostatic interactions with Ad34’s HVR1 loop are rare or absent, at least in its native structural environment. To test this hypothesis, we first constructed Ad5H34, an Ad5-derived vector whose HVR1 loop sequence had been replaced by that of Ad34, and the reciprocal chimeric vector Ad34H5, derived from Ad34 and carrying Ad5 HVR1 loop (Figure 3A). Both Ad34H5 and Ad5H34 displayed significant binding to PF4 in ELISA-qPCR (Figure 3B). Second, we conducted Brownian Dynamics (BD) simulations (Figure S3A) which predicted that close interactions with PF4 were substantially rarer in the case of Ad5-ΔHVR1 and Ad34 than of Ad5 (Figure 3C), fitting experimental data. The similar surface electrostatic profiles between Ad5 and Ad34 (Figure 3D) suggested that whole-hexon surface charge is not the sole parameter determining whether a vector binds PF4 or not. Accordingly, electrophoretic light scattering (ELS) measurements showed that Ad34 and Ad80 had a net surface potential equally or more negative than the PF4-binding Ad types tested (Figure 3E). The models instead pointed toward the HVR1 loop structure as a more likely explanatory factor, with the less protruding HVR1 loop of Ad34 disfavoring interactions (Figure 3F). Moreover, the hexon residues that interacted most frequently with PF4 in BD simulations are clustered in the HVR1 loop, with HVR5 and HVR7 appearing as potential secondary interaction sites (Figure S3B), although the spatial proximity indicated by BD simulations does not necessarily reflect binding affinity. On PF4 proteins, residues of the equatorial ring are predicted to be the ones most frequently involved in interactions (Figure S3C).

Finally, since the Ad5 HVR1 loop is one of the immunodominant epitopes for neutralizing antibodies of Ad5 capsid,^28^ we tested whether PF4 modified Ad5’s susceptibility to neutralizing antibodies. We observed that it reduced Ad5 neutralization levels by human serum intravenous immunoglobulins (Figure 3G).

### Platelet factor 4 binding to adenoviruses modifies their infectivity in multiple immortalized and primary cell types

**Figure 4:**
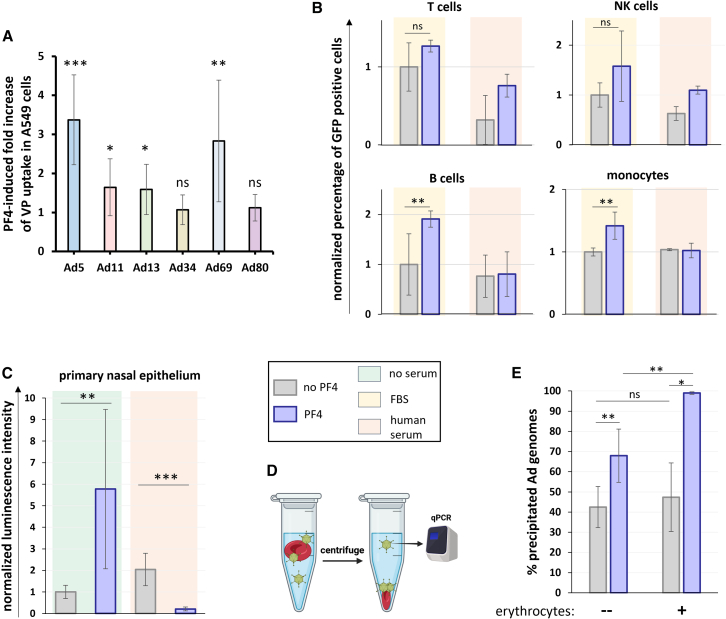
PF4 influences binding Ad infectivity and cell docking with serum and cell type dependency (A–C) For infectivity assays (A–C), VPs were incubated for 10 min at 37°C in optiMEM in the presence or absence of 10 μg/mL PF4, 10% fetal bovine serum (FBS), or human serum seronegative for Ad5, then allowed to infect cultured cells. (A) Fold change in Ad infectivity in A549 cells following Ad incubation with PF4. After infection using 20 vpc, internalized Ad genomes were titrated by qPCR 3 h post-infection (hpi). For each Ad type and each metric, pairwise comparisons were conducted between the samples with and without PF4 using two-sided Mann-Whitney U tests. *N* ≥ 6, two to five independent repeats. Data are represented as mean ± standard deviation. The significance threshold was set at *p* < 0.05. Significance symbols: ns = non-significant, ∗ = *p* < 0.05, ∗∗ = *p* < 0.01, ∗∗∗ = *p* < 0.001. (B) Primary peripheral blood mononuclear cells were infected with 2000 vpc of Ad5 vector from the Ad-GLN collection. Ad-expressed GFP fluorescence was quantified 48 hpi, and the proportions of GFP-positive cells were normalized to the average of the “no PF4, FBS” condition for each cell type. *N* ≥ 3, one or two independent repeats. Data are represented as mean ± standard deviation. Two-sided Mann-Whitney U tests. The significance threshold was set at *p* < 0.05. Significance symbols: ns = non-significant, ∗ = *p* < 0.05, ∗∗ = *p* < 0.01, ∗∗∗ = *p* < 0.001. (C) Primary human nasal epithelium cells were infected with 20 vpc of Ad5 vector from the Ad-GLN collection. Ad-expressed luciferase luminescence was quantified 24 hpi and normalized to the average of the “no PF4, no serum” condition. *N* ≥ 7. Data are represented as mean ± standard deviation. Two-sided Mann-Whitney U tests. The significance threshold was set at *p* < 0.05. Significance symbols: ∗ = *p* < 0.05, ∗∗ = *p* < 0.01, ∗∗∗ = *p* < 0.001. (D) Principle of the erythrocyte pull-down technique. VPs aggregated or docking on erythrocytes, are separated from free VPs by low speed centrifugation and titrated by qPCR. Figure created with BioRender. (E) Erythrocyte pull-down of a fiber-modified Ad5 with ablated CAR tropism (Ad5-ΔCAR) in the absence or presence of PF4. A two-way ANOVA test indicated that both the erythrocytes (*p* = 0.00162) and PF4 (*p* = 1.15E-8) factors were significant. Pairwise comparisons were performed by two-sided Mann-Whitney U tests. *N* = 8. Data are represented as mean ± standard deviation. The significance threshold was set at *p* < 0.05. Significance symbols: ns = non-significant, ∗ = *p* < 0.05, ∗∗ = *p* < 0.01, ∗∗∗ = *p* < 0.001.

Since PF4 modified Ad5 internalization rates in A549 cells even in the absence of human immunoglobulins (Figure 3G), we further studied the impact of PF4 on Ad infectivity. Incubation of Ad5 or Ad69 particles with PF4 increased VP uptake in A549 cells by around 3-fold on average, and of Ad11 or Ad13 by 1.6-fold. On the other hand, PF4 did not influence Ad34 and Ad80 internalization (Figure 4A). PF4 significantly enhanced Ad5 infectivity in A549 cells only at the high concentration of 10 μg/mL (Figure S4A). We extended the Ad5 infectivity assays to a wide array of human immortalized cell lines and primary cells, in the presence or absence of Ad5 seronegative human serum. Extensive differences were observed between cell types and between samples treated with and without serum, and no general rule could be identified regarding the amplitude and direction of the infectivity change induced by PF4 (Figures 4B, 4C, S4B, and S4C). PF4’s effects on blood leukocyte infectivity were modest (Figure 4B), but the permissivity of primary human nasal epithelium was very strongly modified by PF4 both with and without serum, although in opposite directions (Figure 4C). We also tested Ad34 and the closely related but PF4-binding type Ad35 in infectivity assays with primary leukocytes, and PF4 did not substantially affect their infectivity (Figure S4C).

We also tested PF4's effect on Ad binding to erythrocytes, which can result in substantial vector sequestration or retargeting following systemic administrations.^29^ We adapted the APD technique to study docking to human erythrocytes (Figure 4D) of a fiber-modified Ad5 with ablated CAR tropism (Ad5-ΔCAR), whose ability to bind PF4 had been verified by ELISA-qPCR (Figure S1D). PF4 increased Ad5-ΔCAR precipitation to a higher degree in the presence of erythrocytes (Figure 4E), showing that PF4 increased erythrocyte docking. PF4 had no influence on Ad34 docking on erythrocytes (Figure S4D), suggesting that PF4 effects on Ad5-ΔCAR docking depend on direct interactions with VPs and not on potential erythrocyte responses to PF4.

### Platelet factor 4 non-binding COVID-19 vaccine vectors induce robust cellular immune responses in mice

**Figure 5:**
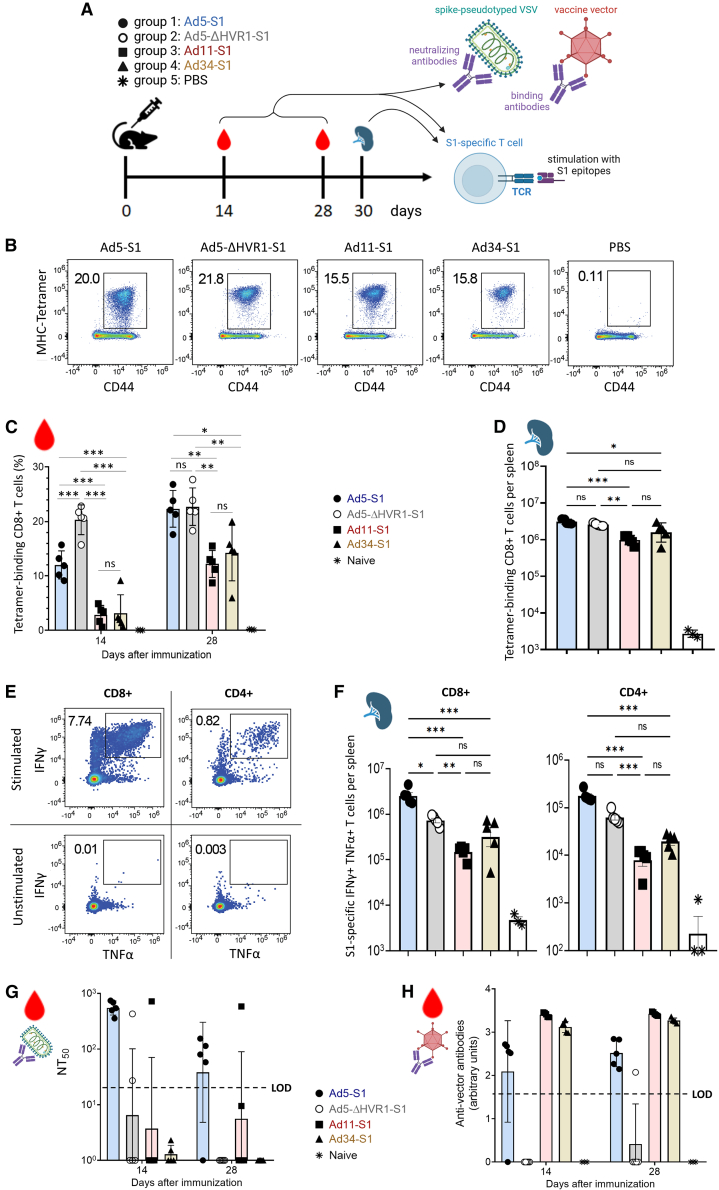
Immunogenicity of PF4 non-binding COVID-19 vaccine candidates in mice (A) Experimental design. Five 10–12 weeks old C57BL/6JCrl male mice per group were immunized intravenously with 5E8 VP of E1-deleted or E1/E3-deleted vectors expressing the S1 domain of the SARS-CoV-2 spike protein (Hu-1 strain). Blood was drawn at 14 and 28 days after immunization (dai), and mice were sacrificed at 30 dai for splenocyte collection. (B–D) (B) Representative FACS plots show the frequencies of peripheral blood S1-epitope specific CD8^+^ T cells in the different groups (C, D). Percentages of S1-epitope specific CD8^+^ T cells in blood (C) and numbers in spleen (D) as determined by MHC tetramer staining. Time-course analysis using a mixed model two-way ANOVA was performed in (C). Data are represented as mean ± standard deviation. Two-sided Mann-Whitney U tests. The significance threshold was set at *p* < 0.05. Significance symbols: ns = non-significant, ∗ = *p* < 0.05, ∗∗ = *p* < 0.01, ∗∗∗ = *p* < 0.001. (E) Representative FACS plots of stimulated or unstimulated IFN-γ- and TNF-α-secreting CD8^+^ and CD4^+^ T cells. (F) Total counts of S1-specific IFN-γ- and TNFα-secreting CD8^+^ (left) and CD4^+^ (right) T cells in spleen upon peptide stimulation. Numbers of IFNγ and TNFα secreting cells in non-stimulated control samples were subtracted. Data are represented as mean ± standard deviation. Two-sided Mann-Whitney U tests. The significance threshold was set at *p* < 0.05. Significance symbols: ns = non-significant, ∗ = *p* < 0.05, ∗∗ = *p* < 0.01, ∗∗∗ = *p* < 0.001. (G) SARS-CoV-2 neutralizing antibody titer (NT50) in mouse serum. Data are represented as mean ± standard deviation. (H) Vector particle binding antibodies in the serum of immunized mice were determined by ELISA. Each serum was tested against the vector used for the immunization of the respective group. Symbols represent individual mice. LOD: limit of detection. Data are represented as mean ± standard deviation.

In order to assess the utility of PF4 non-binding vectors for vaccination purposes, the E1 genes of Ad5-ΔHVR1 and Ad34, as well as their PF4-binding relatives Ad5 and Ad11, were replaced by an expression cassette encoding the SARS-CoV-2 spike protein S1 domain to generate model COVID-19 vaccine vectors. Vector preparations quality was verified by ELS (Figures S5A and S5B), showing expected surface potentials and absence of aggregates, and by transducing units (TU) titration (Figure S5C) that indicated TU/physical titers ratios >5% in A549 cells and consistent within Ad species. Groups of mice were immunized intravenously with 5E8 VP of either one of these Ad vectors or vehicle (Figure 5A). S1-specific CD8^+^ T cells were identified using MHC class I tetramers in the blood of all immunized mice 14 days and 28 days after immunization (Figures 5B and 5C) and in the spleen at day 30 (Figure 5D). Ad5-S1 and Ad5-ΔHVR1-S1 induced the strongest responses, while those elicited by Ad11-S1 and Ad34-S1 were two-to 10-fold lower, depending on the time point and readout. When studying the cells’ differentiation, minor differences between vectors were noted in the early (day 14) expression levels of the effector/memory markers CX3CR1 and CD27 (Figure S6C), but these differences mostly leveled out by day 28 and were not reflected in a differential repartition of S1-specific CD8^+^ T cells into short-lived effector cells (KLRG1+ CD127-) and memory-precursor cells (KLRG1- CD127+; Figures S6A–S6C). Intracellular cytokine staining of day 30 splenocytes showed substantial numbers of polyfunctional IFNγ-/TNFα-coproducing S1-specific CD8^+^ and CD4^+^ T cells (Figures 5E and 5F) with moderate differences between groups reflecting the hierarchy observed in the percentage of S1-specific cells. No major differences between vaccination groups were noted in terms of splenic CD8^+^ T cell effector/memory profiles elicited (Figures S6D and S6F). Taken together, the cellular immune responses induced by Ad5-S1 and Ad5-ΔHVR1-S1 were of similar magnitude and phenotype, whereas those induced by Ad11-S1 and Ad34-S1 exhibited a similar phenotype, too, but were of slightly reduced magnitude. To test the induction of humoral immune responses, we quantified SARS-CoV-2-specific neutralizing antibodies and vector-binding antibodies. Ad5-S1 was the only vector to consistently induce anti-spike neutralizing antibodies, and neutralizing titers decreased by about 10-fold between days 14 and 28 after immunization (Figure 5G). Meanwhile, the anti-vector humoral response was higher in Ad11-S1 and Ad34-S1 vaccinated animals than in mice receiving Ad5 vectors (Figure 5H). In the Ad5-ΔHVR1-S1 immunized group, anti-Ad5-ΔHVR1-S1 antibodies were detectable at low levels in one out of five animals and undetectable in the four others.

## Discussion

Here, we identified the hexon HVR1 loop, already known as the binding site of several Ad5 ligands,^30^^,^^31^ as the most likely binding site of PF4 on adenovirus capsids, at least for Ad5. SPR measurements suggest a model where PF4 is recruited to HVR1 through electrostatic interactions, followed by a more stable hydrophobic interaction, possibly facilitated by HVR1 flexibility, bringing PF4 to another site through an induced fit mechanism. Ad5H34’s binding to PF4 (Figure 3B) may indicate that the precise amino acid sequence of the HVR1 loop plays a less determining role than its flexibility and/or occupancy of the inter-hexon space, which also depends on surrounding hexon sites. On the other hand, the cooperative binding with very slow association kinetics observed for Ad34, Ad80, and Ad5-ΔHVR1 (Figure 2B) may depend on allosteric changes of PF4 conformation and/or the absence of passivating agents and other proteins, and possibly involve HVR5 and HVR7 (Figure S3B). This could explain why this binding was not observed in other assays and conditions applied in this study and although there remains a potential source of residual interaction, this suggests it would not occur at a high frequency in the complex environments of blood and tissues. We therefore consider these vectors as PF4 non-binding Ads. Consequently, they may present a lower risk of thrombosis.

In the present study, we identified Ad types that lack binding to PF4 and show that ChAdY25 and Ad26 vectors with capsids equivalent to the COVID-19 vaccines bind to PF4. The vectors we used are not in the same formulation as Vaxzevria and JCovden, which were applied to humans, but are identical in terms of capsid structure, and we have evaluated PF4 interactions using identical buffers for each vector to ensure comparability. Adenoviral vectors without VITT risk may prevent a number of patients’ deaths and non-fatal complications, and improve public confidence in vaccination. Therefore, we tested the applicability of Ad5-ΔHVR1 and Ad34 as vaccine vectors in mice and detected potent S1-specific cellular immune responses induced by both vectors (Figures 5B–5F and S6). As demonstrated with COVID-19 vaccines, cell mediated immune responses are a main correlate of variant cross-reactive vaccine protection^32^ and are far more durable than antibody titers.^8^ Hence T cells may represent the most important arm of immunity to induce when it comes to the prevention of severe disease.^33^ In terms of humoral responses, only Ad5-S1 induced SARS-CoV-2 neutralizing antibodies (Figure 5G). However, this does not necessarily indicate fundamental immunogenicity differences between vector types, and may be linked to the relatively low vector dose used here (5E8 VP),^34^^,^^35^ or to the S1 subunit antigen which unlike the full-length S protein seems to be a poor inducer of SARS-CoV-2 neutralizing antibodies.^36^ Therefore, the intrinsic difference in immunogenicity between S and S1 may account for the early contraction of the anti-S humoral response observed in immunized mice, and further investigations on the influence of antigen design on adenovirus vectors immunogenicity profile are warranted. Importantly, Ad5-ΔHVR1-S1 induced low anti-vector antibody levels. Since strong pre-existing neutralizing antibody responses against the adenovirus vector vehicle can impede the anti-antigen immune response,^37^^,^^38^^,^^39^^,^^40^ HVR1-deleted vectors may prove beneficial in repeat dose regimens such as homologous prime-boost vaccination, as they would likely not be subject to the same degree of inhibition upon a second administration. In addition, Ad5-ΔHVR1 vectors are less hepatotoxic and less sensitive to serum neutralizing antibodies than wild-type Ad5.^41^ HVR1 deletion in Ad5, the Ad type most frequently used in clinical studies, or other vector types could thus represent a potent and simple engineering method to increase vector safety and facilitate repeated administration. HVR1 polymer shielding may share these advantages, and future developments on the polymer modification of non-Ad5 types will be required to assess the full potential of this method. Furthermore, the potential of Ad34 *in vivo* is supported by its very low seroprevalence^42^ as well as the safety and high biological activity reported for the related species B vectors NG-641 and Enadenotucirev^43^ in clinical trials.^44^^,^^45^ Finally, Ad80 has been predicted to be immunogenic as a vaccine platform^42^ and may deserve consideration.

Continued screening efforts may identify additional AAV and Ad types that lack PF4 binding, to which end the ELISA-qPCR and APD techniques established here could prove valuable. These methods can detect Ad-PF4 interaction despite its relatively low affinity (K_D_ = 1.05 μM for Ad5, Figure S2B), and additional tests should assess their range of applications, which in theory extend to all virus-protein interactions in the case of ELISA-qPCR.

Currently, the most widely accepted hypothesis on VITT etiology is that a subset of pre-sensitized individuals undergoes epitope spreading when exposed to Ad-PF4 complexes, leading to the secretion of highly reactive, platelet-activating anti-PF4 auto-antibodies.^46^ VITT was rarely observed upon the re-administration of PF4-binding adenoviral vectors, presumably because potential anti-PF4 antibody responses are likely to be overwhelmed by the polyclonality of the anti-vector response. However, exposure to different PF4-binding vectors or viruses sharing few epitopes other than vector-bound PF4 may lead to a boosting and expansion of pathogenic auto-antibodies and increase the risk of thrombosis. This presents the possibility that patients were VITT were pre-sensitized by an earlier infection with a PF4-binding virus, and that a large number of COVID-19 vaccinees would now be primed for an acute secondary immune response and potentially thrombosis in case of future exposure to PF4-binding vectors. In this case, there would be a crucial medical need for the development of PF4 non-binding viral vectors.

Besides its involvement in VITT, PF4 binding modified Ad infectivity (Figures 4A–4C) and may increase Ad sequestration on erythrocytes (Figure 4E), a known cause of vector inactivation and toxicity,^29^^,^^47^ but on the other hand could protect vectors from neutralizing antibodies (Figure 3G). Altogether, PF4 binding may modify the tropism of clinical Ad vectors. Although representative animal models of VITT are not available due to the rarity of this syndrome, mice may be a suitable model to study non-thrombotic consequences of adenovirus binding to PF4 since the viruses that bound to mouse PF4 also bound to human PF4 and vice-versa (Figure 1F).

Even though the AAV-associated TMA syndrome^22^ differs from the Ad-related VITT in its clinical presentation, it is striking that the AAV9 type associated with most identified cases binds to human PF4.^27^ Furthermore, pathogens such as HIV-1 are able to bind to PF4^48^ and associate with deep vein thrombosis and thrombocytopenia,^49^^,^^50^ and a VITT case was reported following papillomavirus vaccination.^51^ Thus, investigations on other vectors and virus families for interactions with PF4 and the potential pathologic consequences are warranted.

### Limitations of the study

Due to the rarity of VITT events and the current lack of appropriate animal models, we cannot claim that vectors that do not interact with PF4 are, *per se,* safer than PF4-interacting counterparts. However, we can reasonably assume that a reduced number of interactions with host serum and cell components would increase the level of control, reproducibility, and safety of Ad vectors in the clinic. Further investigations on VITT etiology may also facilitate the establishment of animal or *in vitro* models to test whether vaccine platforms activate early steps of the VITT causal chain. Note that male mice were used for the experiments reported in this article, and the potential influence (or association) of sex, gender, or both on the results of this animal study has not been investigated, which may represent a limitation to the generalizability of the results obtained. Downstream experiments remain to be performed investigating factors involved in the thrombotic phase of VITT. For instance, the immunodominant epitopes in PF4 and how their modulation might impact VITT could be analyzed by PF4 mutants.^52^ These mutants may also have served as specificity controls in our assays; however, since biologically active PF4 is difficult to produce in sufficient quantities, we have refrained from generating structure-based PF4 mutants. Instead, TadA and coagulation factor X (both with and without calcium) were used to assess the specificity of observed PF4 binding, since they share a similar size and a similar surface charge with PF4. We observed that ELISA-qPCR recapitulated known results concerning Ad binding (Figures S1A and S1B), confirming specificity.

Our study of PF4 binding to Ad vectors and its effects on infectivity is focused on situations where platelets are activated, and PF4 concentration is high, which can be expected to frequently occur locally in vaccinees due to inflammation or minor wounds. Our dose response experiment (Figure S4A) indicates that infectivity changes are likely negligible at physiological PF4 concentrations (<0.1 μg/mL), although significant effects could be expected in intermediate PF4 concentrations for certain cell types, such as epithelial cells (Figure 4C) or erythroid progenitors (Figure S4B).

Finally, measurements of Ad vectors binding to or infection of human primary cells were conducted with a limited number of donors, and it is thus possible that inter-individual differences exist in the described phenotypes. This, however, does not infirm the main conclusions of these experiments, namely, which Ad types bind or do not to PF4, and that PF4 can strongly modify Ad infectivity levels in a variety of cell types.

In conclusion, we identified several vectors, namely AAV1, AAV6, AAV7, Ad34, Ad80, and Ad5-ΔHVR1, as lacking PF4 binding. Ad34 and Ad5-ΔHVR1 in particular showed applicability and non-inferiority as preclinical vaccine platforms. These results may represent a milestone in the development of safer Ad vectors.

## Resource availability

### Lead contact

Requests for further information and resources should be directed to and will be fulfilled by the lead contact, Anja Ehrhardt (anja.ehrhardt@uni-wh.de).

### Materials availability

This study did not generate new unique reagents.

### Data and code availability

This article does not report original code. All data associated with this study are present in the article or the supplemental information. Any additional information required to reanalyze the data reported in this article is available from the lead contact upon request.

## Acknowledgments

We are grateful to Dr. Inga Seuthe (Sankt-Johannes Hospital, Hagen, Germany) and Merve Torun (then working at Witten/Herdecke University, Witten, Germany) for providing and maintaining the clinical samples used for nasal epithelium culture, and to Prof. Rob C Hoeben for initiating and coordinating the isolation of Goravir.

Funding: 10.13039/501100001659DFG grant EH 192/5-3 (A.E.); PhD program Witten/Herdecke University (E.Sa., D.W.).

## Author contributions

Conceptualization: E.Sa., V.C., D.Pi., F.K., and A.E. Methodology: E.Sa., D.Pe., L.M.B., F.H., S.F., W.B., M.A., V.K., A.L.P., D.G., T.V., A.T.B., W.Z., D.Pi., F.K., and A.E. Investigation: E.Sa., D.Pe., M.C., L.M.B., D.W., F.H., F.J., E.C., S.S., N.B., K.S., M.T., G.K., X.W., and N.S. Formal analysis: E.Sa., D.Pe., M.C., L.M.B., C.K.C., E.C., E.Sc., D.S., T.V., A.S., and A.T.B. Writing – original draft: E.Sa. Writing – review and editing: E.Sa., D.Pe., M.C., D.W., S.S., K.S., G.K., X.W., W.B., M.A., A.T.B., W.Z., D.Pi., F.K., and A.E. Funding acquisition: A.T.B., V.C., D.Pi., F.K., and A.E.

## Declaration of interests

The authors declare no competing interests.

## STAR★Methods

### Key resources table

**Table:** 

REAGENT or RESOURCE	SOURCE	IDENTIFIER
**Bacterial and virus strains**		

E. coli cells propagating the Red/ET expressing plasmid	Gene Bridges	K001
*E. coli* strain GB05-dir	Gene Bridges	K008
adenovirus vectors	Zhang et al.*,*^25^ Bots et al.*,*^26^ this study	N/A
adeno-associated virus vectors	Weinmann et al.^53^	N/A

**Chemicals, peptides, and recombinant proteins**		

Human PF4	Chromatec	PF4-h
Spike protein peptide pool	GenScript	RP30020

**Critical commercial assays**		

my-Budget 5x EvaGreen qPCR-Mix II	Bio-Budget	*80-59XXXXX*

**Experimental models: organisms/strains**		

C57BL/6 wt mice	Charles River laboratories	N/A

**Oligonucleotides**		

GLN-for	accaagcgaaacatcgcatcgag	vectors of the Ad-GLN collection
GLN-rev	gcgataccgtaaagcacgaggaag	vectors of the Ad-GLN collection
WT-for	GCCCCAGTGGTCTTACATGCACATC	vectors of the Ad-WT collection
WT-rev	GCCACGGTGGGGTTTCTAAACTT	vectors of the Ad-WT collection
WT-probe	CCGGGTCTGGTGCAGTTTGCCCGC	vectors of the Ad-WT collection
CMV-for	tacatcaatgggcgtggata	vaccine-equivalent vectors
CMV-rev	ggcggagttgttacgacatt	vaccine-equivalent vectors
Fiber-for	accggtttccgtgtcatatgg	Ad5 hexon mutants and Ad5-ΔCAR
Fiber-rev	ggtattgcagcttcctcctgg	Ad5 hexon mutants and Ad5-ΔCAR
AAV-for	aacgccaatagggactttcc	vectors of the AAV collection
AAV-rev	gggcgtacttggcatatgat	vectors of the AAV collection
cell-for	GGAATTGATTTGGGAGAGCATC	human beta-2-microglobulin
cell-rev	CAGGTCCTGGCTCTACAATTTACTA	human beta-2-microglobulin
cell-probe	GAAGGTGGATGATCTGCCCAGTCACACT	human beta-2-microglobulin

**Recombinant DNA**		

mouse PF4 cDNA	SinoBiological	MG50144-M

**Software and algorithms**		

SimpleARBD	This study	N/A

**Other**		

HiTrap Heparin HP columns	Cytiva	17040601
high-binding ELISA plate	Sarstedt	82.1581.200
MP-SPR 2201A instrument	Bionavis Ltd	N/A
Zetasizer Advance Serie – Ultra Red	Malvern Panalytical	N/A

### Experimental model and study participant details

Human donors: The studies were approved by the ethics committee of the University Witten/Herdecke. Approval numbers: 159/2022, October 10th 2022; 209/2020; 216/2020, December 17th 2020. All relevant ethical regulations have been followed and all donors gave informed consent; due to anonymysation requirements, the age, sex, ethnicity or other donor information cannot be disclosed.

#### Mouse study

Origin of animals: Charles River laboratories; Species and strain: C57BL/6JCrl mice; Sex and husbandry: male, 10-12 weeks-old, specific-pathogen-free conditions, 22°C.

Ethics approval: Animal experiments were performed at the University of Basel in accordance with the Swiss law for animal protection and with permission by the Cantonal Veterinary Office of Basel City (permission #35138/2666).

### Method details

#### Cell culture

Cells were cultivated with Dulbecco’s Modified Eagle Medium (DMEM, Pan-Biotech; for A549, CaCo2, EaHy926, HEK293, HEK293T, Hela, Hep3B, HepG2, MiaPaCa2 and SkBr3 cells) or Roswell Park Memorial Institute 1640 Medium (RPMI, Pan-Biotech; for HCC827, Jurkat, K562, SKOV-3 and THP-1 cells), each supplemented with 10% (or 20% in the case of CaCo2 and HCC827 cells) Fetal Bovine Serum (FBS, Pan-Biotech) and 1x Penicillin-Streptomycin (P/S, Pan-Biotech) at 37°C under an atmosphere with 5% CO_2_. THP-1 cells were additionally supplemented with 50 μM β-mercaptoethanol. THP-1 macrophages were differentiated 72h in presence of 20 ng/mL of phorbol-12-myristat-13-acetate (PMA). Cells were tested for mycoplasma infection using the VenorGeM OneStep kit (Minerva Biolabs).

Peripheral blood mononuclear cells (PBMCs) were isolated from the blood of healthy adult volunteers collected in EDTA tubes using SepMate tubes (Stemcell technologies) following manufacturer’s instructions. PBMCs were counted and cultivated in RPMI + 10% FBS + P/S media for 24 hours in low adherence dishes (Corning, #3471) before infection. The study was approved by the ethics committee of the University Witten/Herdecke (approval number 159/2022, October 10^th^ 2022; all relevant ethical regulations have been followed and all donors gave informed consent; due to anonymysation requirements, the age, sex, ethnicity or other donor information cannot be disclosed).

Nasal epithelium cultures were derived from patients’ samples with chronic rhinosinusitis and nasal polyposis undergoing an operational procedure. Tissue samples were first cut into small portions and placed in collagenized cell culture flasks (Greiner Bio-One, AT) filled with BEGM® medium (Lonza, Switzerland). Portioned tissue pieces were then removed (usually after 1-3 days) from the cell culture flasks and the culture was continued using the outgrowth technique as described previously.^54^ After two to three weeks, a 90% confluence rate was achieved and the culture purity was assessed by flow cytometry using epithelial cell-specific antibodies as described previously. The cells were then seeded into collagenized cell culture plates and used for infection assays. The sample collection and obtaining of consent was conducted under strict observance of relevant ethical regulations and under a positive ethics approval from the Witten/Herdecke University, Germany (approval number: 209/2020; due to anonymsation requirements, the age, sex, ethnicity or other donor information cannot be disclosed).

#### Vector acquisition

The Ad-WT collection has already been described in Wang et al.^42^ The Ad-GLN collection, already described in Zhang et al.*,*^25^ contains vectors from different types that express TurboGFP, NanoLuc luciferase and the selection marker kanamycin/neomycin under a synthetic CAG promoter in the deleted E3 region. The CAG promoter consists of the human cytomegalovirus early enhancer element, the chicken beta-actin promoter including parts of the first exon and intron, and parts of the second intron and third exon of the rabbit beta-globin gene. The gorilla adenovirus oncolytic vector Goravir was constructed as described in Bots et al.^26^

The hexon-modified Ad5 vectors were produced similarly as described elsewhere.^55^ Briefly, genetic capsid modifications were introduced using pRed/ET homologous recombination (Gene Bridges, Heidelberg, Germany) in a bacmid carrying an Ad5 genome (AY339865) with the E1 locus (bp 441-3534) replaced by a CMV-promoter driven eGFP expression cassette. Vector PEGylation was conducted with 5 kDa mono-activated maleimide polyethylene glycol as described elsewhere.^55^

The CAR-ablated Ad5 (previously described^56^) was generated by introducing the point mutation Y477A (AAQ19310.1) in the fiber gene of the GFP-expressing, E1-deleted Ad5 backbone with the pRed/ET recombination kit. The Ad5F35 vector contains Ad35 fiber (positions 30954 to 31794) replacing the original Ad5 fiber (positions 31169 to 32782). The Ad5-ΔHVR1 vector was constructed by incorporating the T425A hexon mutation which ablates binding to factor X, and substituting the 22 amino acids at the positions 141 to 162 of the hexon protein by the neutral peptide GGSG, as described elsewhere.^41^ The Ad5H34 vector was constructed from the Ad5-ΔHVR1 as backbone using the pRed/ET recombination kit, by substituting the 22 amino acids at the positions 141 to 162 of Ad5’s wild-type hexon for the 17 amino acids at the positions 141 to 157 of Ad34’s hexon protein. Likewise, Ad34’s hexon amino acids 141 to 157 were replaced by Ad5’s hexon amino acids 141 to 162 by recombineering^25^ to generate the Ad34H5 vector.

To generate the model S1 vaccine vectors, the human codon-optimized S1 domain of SARS-CoV-2 Wuhan strain (pUC57-2019-nCOV-S plasmid, GenScript Biotech Corporation) was subcloned under a CMV promoter and in front of a SV40 poly-A terminator. This expression cassette was then inserted in left-to-right orientation in *lieu* of the E1 gene (genomic positions 441 to 3521) of an E3-deleted Ad5 genome, as well as Ad11 (468 to 3271) and Ad34 (469 to 3272) backbones carrying Ad5 E4–ORF6 (PCR-amplified from Ad5 genomic positions 32958 to 34072) instead of their own homologous ORFs (genomic positions 31875-32973 for Ad11 and 31861-32959 for Ad34) so that the resulting Ad11-S1 and Ad34-S1 vectors could be rescued in HEK293 cells despite their lack of E1 gene. The Ad5-ΔHVR1-S1 vector was obtained from Ad5-S1 by substituting the 22 amino acids at the positions 141 to 162 of the hexon protein by the neutral peptide GGSG.

All adenovirus vectors produced for this study, including ChAdY25 and Ad26 vectors with capsids equivalent to the COVID-19 vaccines, were grown on HEK293 cells and purified by double CsCl banding and subsequent desalting with PD-10 columns (SE Healthcare). Ad vectors were titrated by optical density measurements^56^ and silver staining of VP proteins after polyacrylamide gel electrophoresis in reducing conditions.

Adeno-associated vectors were produced, purified and titrated as described previously.^53^

#### Mouse PF4 purification

Mouse PF4 cDNA (SinoBiological #MG50144-M) was cloned under a CMV promoter with CMV enhancer and in front of a bGH poly-A terminator in a pcDNA3.1-C-(k)DYK plasmid backbone (Table S1). The mouse PF4 expression plasmid was transfected in HEK293T cells using the jetOPTIMUS reagents (Polyplus #101000006) following manufacturer’s instructions. At 3 hours post transfection, cells were washed twice and cultivated in Ex-cell media (Merck #14571C) supplemented with P/S and GlutaMAX (Gibco #35050038). Four days later, media was harvested, centrifugated at 300g for 3 min and the supernatant was treated with 2 protease inhibitor tablets (Roche #11697498001) then centrifugated at 300g for 3 min again then at 18,000g for 30 min at 4°C. The supernatant was filtered (0.2 μm pores, Sarstedt #83.3940.101) and the buffer was exchanged for desalting buffer (20 mM Tris-HCl, pH=7.80) using a PD-10 column (Cytiva #17085101). Secreted mouse PF4 was then purified by FPLC using a BioRad NGC Quest Plus chromatography system and HiTrap Heparin HP columns (Cytiva #17040601) as followed: the column was washed with 10 column volumes (CV) of desalting buffer, then the sample was loaded, the column was washed with 10 CV desalting buffer, then a linear salt gradient was applied up to 2M NaCl in 10 CV to elute mouse PF4. Elution fractions were collected and their purity was verified by silver staining: aliquots were denatured using Laennli buffer for 10 min at 80°C then loaded on a 15% polyacrylamide gel under reducing conditions, which after electrophoresis was stained as described previously. Protein concentration was measured by Quick Start Bradford assay (BioRad) following manufacturer’s instructions.

#### ELISA-qPCR

Proteins used in this study were human platelet factor 4 (PF4-h, Chromatec), which was stored at 4°C in PBS at a concentration of 200 μg/mL; mouse platelet factor 4; human factor X (fX, Cellsystems #HCX-0050-MG); S.typhimurium tRNA-specific adenosine deaminase (tadA, MyBioSource #MBS1445221); and soluble CAR receptors (sCAR, SinoBiological, #10799-H08H). Proteins of interest were diluted in coating buffer (0.1 M NaHCO_3_, pH set between 9.2 and 9.6 using 1 M Na_2_CO_3_) to a concentration of 20 μg/mL and 75 μL were added per well of ELISA plate (Sarstedt #82.1581.200), which was sealed with a transparent film and incubated overnight at 4°C. Wells were washed twice with TBS-Tween (TBST; 0.5% Tween20), blocked with blocking buffer (TBST + 0.5% pork skin gelatin) for 1 h at room temperature (RT), and washed twice with TBST. Vector particles were diluted in blocking buffer and incubated in the chosen coated wells for 2 h at 37°C. Except stated otherwise, 5E7 VP of adenovirus vectors or 5E8 VP of adeno-associated vectors were used per well with 75 μL total volume. After virus incubation, wells were washed four times with TBST in order to eliminate VPs which did not interact specifically with the coated proteins. To quantify the remaining VPs, 75 μL of alkaline lysis buffer (25 mM NaOH + 2 mM EDTA) were added per well and the plate was carefully and tightly sealed and heated at 95°C for 10 min. to open capsids and release vector genomes. The plate was then immediately put on ice and 25 μL of cold neutralization solution (80 mM Tris-HCl + 0.1% Tween20, pH = 3.2) were added in each well. The virus genome solutions of each well were homogenized by shaking and two 2 μL aliquots were taken for qPCR titration using a CFX96 Real-Time System machine (BioRad) and the my-Budget 5x EvaGreen qPCR-Mix II (Bio-Budget) following the manufacturer’s instructions, except for vectors of the Ad-WT collection which were quantified using Takyon No ROX Probe 2x MasterMix dTTP blue (Eurogentec) and the universal hexon primer/probe set of Heim et al*.*^57^ See Table S2 for primers. Since Ad vectors used for ELISA-qPCR were purified by cesium chloride ultracentrifugation, enabling the elimination of empty VPs, the genomic titers measured by qPCR exactly correspond to VP titers.

#### Aggregate pull-down

1E7 VPs were incubated for 30 min. at 37°C in 30 μL of PBS + 1% BSA with or without 10 μg/mL of PF4. After centrifugation for 5 min. at 1000 g, the supernatant was transferred to a new tube containing 30 μL of 2x alkaline lysis buffer (50 mM NaOH + 4 mM EDTA), while 30 μL of 2x alkaline lysis buffer and 30 μL of PBS + 1% BSA were added to the pellet. Both treated supernatant and pellet were mixed thoroughly and heated at 95°C for 10 min. in order to release Ad genomes, then neutralized with 20 μL of cold neutralization solution and titrated by qPCR (see Table S2 for primers).

#### Surface plasmon resonance

For Figure 2A, a BIAcore T200 (Cytiva, formerly GE Healthcare) equipped with a C1 sensor chip (Biacore) was used to generate binding profile at 25°C in a running buffer of HBS-EP+ [10 mM HEPES, 150 mM NaCl, 3 mM EDTA, and 0.05% (v/v) Surfactant P20]. To prepare the capture surface, viruses were amine-coupled under standard conditions at a flow rate of 10 μL/min, as follows. Each flow cell was activated with a freshly prepared 1:1 v/v mixture of aqueous stocks of 0.4 M 1-ethyl-3- (3-dimethylaminopropyl) carbodiimide (EDC) + 0.1 M N-hydroxysuccinimide (NHS) for 240 s. Viruses were diluted to ∼2.5 × 1E10 VP/ml in 10 mM acetate 3.5 buffer and coupled for 30 min. Finally, excess reactive esters were blocked with 1 M ethanolamine-HCl pH 8.5 for 1 min. Human PF4 was prepared in PBS + 0.5% BSA + 0.005% P20 at nominal concentrations of 0, 5.9, 11.7, 23.4, 46.9, 93.8, 187.5, 375, 750, 1500 and 3000 nM and injected over all flow cells for 90 s at a flow rate of 10 μL/min. All sensorgram plots were subtracted from the reference flow cell to remove the nonspecific responses, bulk refractive index changes, and systematic instrument noise.

For Figures 2B and S2, an MP-SPR 220A instrument (Bionavis Ltd, Tampere, Finland) equipped with a 2D carboxymethyldextran (CMD 2D) sensor was used to measure the binding profiles at 22°C. Viruses were immobilized onto the CMD 2D surface following standard amino coupling methods. Briefly, sensors were activated with a 7 min. 0.05M N-hydroxysuccinimide (NHS)/0.2M N(-3-dimethylaminopropyl)-N-ethylcarbodiimide hydrochloride (EDC) injection. Viruses were diluted to 1E11 VP/mL in 50 mM 2-(N-morpholino) ethane sulfonic acid (pH 5.0). Unreacted succinimide esters were inactivated with a 5 min. injection of 1M ethanolamine (pH 8.5). Surfaces were purified with two 2 min. injections of 10mM NaOH, followed by an exchange of running buffer to PBS. PF4 was injected in PBS at 7 μg/mL for 10 min., then flushed 10 min. with PBS. Sensors were regenerated by two 3 min. injections of 2M NaCl + 0.01M NaOH.

#### Structural modellings and MD simulation

The structural model PDB 6B1T (Ad5 hexon) was employed and compared against the corresponding amino acid sequence from GenBank to identify segments of missing residues. Sequences of these segments of missing residues, termed missing peptides here, were fed to AlphaFold (Web Services) to generate corresponding tentative structures. These missing peptides were then manually adjusted and positioned back to either Ad5 so that the position of the beginning residue of each peptide was within 8 Å of the residue preceding it in the corresponding amino acid sequence and the ending residue of each peptide was within 8 Å of the residue after it in the corresponding amino acid sequence. The structure model of Ad34 hexon was generated entierely from AlphaFold (Web Services).

Molecular dynamics (MD) simulations were applied on the above starting models for Ad34 and Ad5 to prompt each of the grafted missing peptides adopting a conformation that would be in equilibrium with the rest of the hexon trimeric complexes. To do so, 25 hexons were first laid out tightly onto a plane to mimic the tight-packing environment of hexons over the capsid (Figure S3A). These ensembles of hexons were then simulated with explicit solvent for 4 μs under a hexagonal periodic boundary condition to equilibrate the starting hexon modes for Ad34 and Ad5. The same model building and simulation strategy were applied to Ad5-ΔHVR1. PF4 tetramer structure was adopted from PDB 1RHP.

#### Brownian dynamic simulations and contact analyses

The last frames of the respective MD simulations for each hexon ensemble were used as the representative structural model for the considered adenovirus. The corresponding mean field descriptions were derived following previous work^18^^,^^58^^,^^59^ using an in-house python module, SimpleARBD (unpublished, Github) and included the charge-electrostatic representation and the contact force profiles. The mean field representations were fed into the Atomic Resolution Brownian Dynamics (ARBD) simulation engine for Brownian dynamic (BD) simulations as previously described.^18^^,^^58^^,^^59^^,^^60^ The initial setting for the corresponding BD simulation was to lay the hexon ensemble on the x-y plane with the ensemble’s center of mass being located at the origin. Then, 400 copies of PF4 tetramers were scattered randomly on a plane parallel to the x-y plane and was 150 Å vertically above the outermost surface of the hexon ensemble (Figure S3A) and their diffusion was simulated for 4 μs. PF4 tetramers were not interacting with one another during the simulations, thus mimicking the docking process of PF4 from a diverse pool of initial locations in the bulk outside the adenovirus’ capsid. The distance between each copy of PF4 and the hexon ensemble as well as their contacts were monitored and analysed by the analysis module of SimpleARBD. In this study, a copy of PF4 was considered making contacts with the hexon ensemble if any of the PF4’s heavy atoms came within 5 Å of the heavy atoms of the hexon ensemble.

#### Electrophoretic light scattering measurements

To ensure homogeneous measurement conditions and best visibility of Zeta(ζ)-potential changes, vectors were submitted to buffer exchange prior to ELS measurement. Buffer exchange was performed using 5E10 VPs and PD-10 MiniTrap G-25 columns (GE Healthcare, Solingen, Germany) following the company’s instructions with the “gravity” protocol. Briefly, the column was equilibrated using the measurement buffer (50 mM HEPES, pH 7.2). Afterwards, vectors were added to the column and subsequently, the vector volume was adjusted to 500 μL by adding measurement buffer to the column. The column was placed on a 1.5 mL reaction tube and the vector was eluted using 1 mL measurement buffer. Complete sample volume was used to measure “particle concentration” (Zetasizer Advance Serie – Ultra Red, Malvern Panalytical, Kassel, Germany) in a glass cuvette with square aperture (PCS1115). Thereby, size and concentration were determined by multiple angle dynamic light scattering (MADLS) with three measurement repeats (25°C, dispersant scattering mean count rate 179 kcps, dispersant values: R.I. 1.33; viscosity 0.8872 mPa s). For ζ-potential measurement, 700 μL of the suspension were transferred to a folded capillary cell (DTS1070). To ensure sample integrity, three size measurements in backscatter mode where done before and after the ζ-potential measurement (25°C, dispersant values as given above). ζ-potential measurement was done in “general purpose” mode with a minimum number of runs of 10, three repeats and 60 seconds pause between each repeat (25°C, dispersant values as given above).

#### Infectivity assays

To test the impact of PF4 on the infectivity of Ad-GLN collection vectors in immortalized cells, 2E7 VP/mL were incubated 10 min. at 37°C in OptiMEM (Gibco) with or without 10 μg/mL of PF4. In certain experiments, 10% human serum collected in serum tubes (Sarstedt 01.1601) from a healthy volunteer seronegative for Ad5 and Ad34 were added to the incubation mix. At the end of the incubation time, the culture medium of subconfluent cells was replaced by the virus suspension, resulting in 20 VP/cell (vpc). At 3 hours post infection (hpi), cells were either washed three times and harvested to titrate internalized Ad genomes, or the infection suspension was replaced by culture media and cells were kept in culture until early Ad gene expression was measured by luciferase assay or flow cytometry.

To quantify early infection rates (Figures 4A and S5C), cell DNA was extracted using the Monarch genomic DNA purification kit (NEB #T3010L) or the NucleoSpin Tissue kit (Macherey-Nagel #740952-250) following manufacturer’s instructions. Internalized Ad genomes and cell genomes were titrated by qPCR and the number of Ad genomes per cell was used as an estimator of infectivity (see Table S2 for primers).

Luciferase luminescence (Figures 3G, 4C, S4A, and S4B) was measured 24 hpi using the Nano-Glo® Luciferase Assay (Promega, Madison, USA) kit, a TECAN infinite f plex plate reader and black 96-well luciferase plates (Thermo Fisher Scientific Nunc A/S).

GFP fluorescence intensity (Figure S4C) was measured 24 hpi. Cells were harvested, washed twice in PBS, fixed 10 min. in 2% formaldehyde, washed twice in PBS then resuspended in PBS and analysed by flow cytometry (CytoFlex, Beckman Coulter, Munich, Germany) in FITC channel (585/42 nm), excited with a 488 nm laser.

Infectivity assays on PBMCs were conducted similarly to immortalized cell lines except that 2000 vpc were used, VP incubation and infection was not conducted in optiMEM but in RPMI media supplemented with either 10% FBS or 10% human serum, and Ad-expressed GFP fluorescence intensity was measured by flow cytometry at 48hpi. To this end, cells were washed twice in PBS + 0.5% bovine serum albumin (BSA), Fc receptors were blocked by incubation at 4°C for 15 min. in BD Horizon Brilliant stain buffer (BD Biosciences) + 6% human Trustain FcX solution (BioLegend). Cells were then splitted in two equal groups and stained for 25 min. at 4°C with either anti-CD45 (BioLegend #368524), CD8a (BD Pharmingen #555369), CD3 (BD Biosciences #562426) and CD56 (BD Biosciences #557747), or anti-CD45, CD20 (BD Biosciences #340908), CD14 (BioLegend #301830) and CD11b (BioLegend #301322) fluorophore-coupled antibodies to enable cell type identification. Cells were washed twice more in PBS + 0.5% BSA, incubated for 5 min. at 4°C in BD Horizon Brilliant stain buffer + 4% of 7AAD dye in order to stain dead cells. The samples were then diluted 1:1 in PBS and analysed by flow cytometry.

#### Erythrocyte pull-down

This assay was inspired from Carlisle et al.^29^ Venous blood was collected into EDTA tubes (Sarstedt, 02.1066.001) from the antecubital vein of a healthy adult volunteer who gave informed consent. The blood was swiveled at room temperature (RT) for 15 min. then centrifuged for 5 min. at 2000 g in order to isolate erythrocytes, which were then washed three times with PBS, resuspended in PBS + 1% BSA to a concentration of 5.5E9 cells/mL, and kept at 4°C for no more than 3 days. 4E7 VPs were incubated 30 min. at 37°C in 80μL of the erythrocyte suspension with or without 10 μg/mL of PF4. The suspension was centrifuged for 5 min. at 1000 g and the supernatant was transferred to a new tube. Both supernatant and pellet were treated with 2x alkaline lysis buffer, mixed thoroughly and heated at 95°C for 10 min. in order to release Ad genomes, then neutralized with neutralization solution and titrated by qPCR (see Table S2 for primers).

The study was approved by the ethics committee of the University Witten/Herdecke (approval number 216/2020, December 17^th^ 2020; all relevant ethical regulations have been followed and all donors gave informed consent; due to anonymysation requirements, the age, sex, ethnicity or other donor information cannot be disclosed).

#### Mice and animal experimentation

C57BL/6 wt mice were purchased from Charles River laboratories and were kept under specific-pathogen-free conditions for colony maintenance and experiments. Experimental groups were sex- and age-matched. Mice were bred at the ETH Phenomics Center Zurich (EPIC), whereas experiments were performed at the University of Basel in accordance with the Swiss law for animal protection and with permission by the Cantonal Veterinary Office of Basel City (permission #35138/2666). For adenoviral vector immunization, 5E8 VP were administered in a volume of 200 μl into the tail vein. The intravenous route was used here since the resulting variability in immune responses within a given group is smaller than after intramuscular administration, as observed in observed in head-to-head comparisons using ChAdOx1 and MVA vaccine vectors.^24^^,^^61^

Blood samples were stained immediately after collection with antibodies against CD45R/B220 (RA3-6B2), CD8 (53-6.7), CD44 (IM7), CD62L (MEL-14), CD127 (A7R34), Klrg1 (2F1), CX3CR1 (SA011F11), CD27 (LG3A10) and CD43 (1B11) purchased from BioLegend and subsequently treated with FACS lysing solution (BD Biosciences, Cat. #349202) to remove erythrocytes and fix the cells. For detection of S1-specific CD8^+^ T cells, H2-K^b^ tetramers were conjugated to PE and loaded with the SARS-CoV-2 spike epitope (VNFNFNGL) by the University of Lausanne Tetramer core facility. The tetramers were added to the antibody mix for staining. Spleens were mechanically disrupted and counted with an Immunospot S6 device (C.T.L.). For surface staining, splenocytes were incubated with the same cocktail of antibodies and tetramer as used for blood with the addition of anti-erythroid cells antibody (TER-119). Dead cells were stained with Zombie-NIR Fixable Viability Kit (BioLegend, Cat: #423105). Samples were fixed by incubation with 2% paraformaldehyde for 15 minutes at room temperature. For functional assays, splenocytes were restimulated with overlapping peptide sets spanning the spike protein purchased from GenScript (Cat. #RP30020) and stained by intracellular cytokine assays as described previously.^62^ In addition to anti-CD45R/B220 and CD8, antibodies against CD4 (RM4-5), IFN-γ (XMG1.2) and TNF-α (MP6-XT22) were used. All samples were measured on a 5-laser Aurora spectral flow cytometer (Cytek Biosciences, Fremnont, CA, USA) and analyzed with FlowJo Software (BD Biosciences).

#### Neutralizing antibody assays

To identify seronegative donors, human blood was collected in serum tubes (Sarstedt 01.1601) from healthy volunteers (the study was approved by the ethics committee of the University Witten/Herdecke with the approval number 159/2022, October 10^th^ 2022; all relevant ethical regulations have been followed and all donors gave informed consent), which were inverted several times and centrifugated at 2,000 g for 10 min. The serum supernatant was heated at 56°C for 30 min. and diluted 1:10 in pure DMEM. Twofold dilution series up to a serum dilution of 1:2560 were then performed using DMEM + 10% FBS as diluent in order to equalize the total serum concentration, then incubated for 1h at 37°C with 5E7 VP/mL of the chosen vector of the Ad-GLN collection. DMEM + 10% FBS was used as control. The incubation mix was then distributed onto subconfluent A549 cells, resulting in 100 VP/cell (vpc). Media was changed 3 hours post infection (hpi) and luciferase luminescence was measured 24 hpi using the Nano-Glo® Luciferase Assay (Promega, Madison, USA) kit, a TECAN infinite f plex plate reader and black 96-well luciferase plates (Thermo Fisher Scientific Nunc A/S). Sera showing no decrease in luminescence signal even at 1:10 dilution compared with the FBS control were considered seronegative.

To assess the effects of PF4 in presence of Ad5-neutralizing antibodies, Ad5 VPs from the GLN collection were incubated with or without 10 μg/mL PF4 in OptiMEM for 30 min. at 37°C. Meanwhile, IVIGs were diluted from 1:5 to 1:400 in FBS, human seronegative serum was diluted 1:5, and an FBS-only sample was prepared for the untreated (“UT”) control. All of these samples were heat-inactivated for 30 min. at 56°C then added to the vector suspensions at 10% final volume. Following further 15 min. incubation at 37°C, the suspensions were added onto subconfluent A549 cells at 500 vpc and luciferase assays were conducted at 24 hpi as described above.

SARS-CoV-2 neutralizing antibodies in blood were measured by diluting serum samples from naive or immunized mice in MEM + 2% FCS, starting with a 1:10 dilution followed by three-fold serial dilutions in 96-well plates. Each dilution of serum or monoclonal antibody S309,^63^ serving as a positive control, was incubated with an equal volume of replication-deficient rVSV-EGFP pseudotyped with SARS-CoV-2 spike protein (Wuhan Hu-1 strain) containing approximately 100 infectious units for 1 hour at 37°C. Subsequently, the mixture was incubated with Vero E6 cells (2x10^4^ cells/well) for 16 hours and fixed with 2% paraformaldehyde. The number of green spots was quantified using an Immunospot S6 device (C.T.L.). The 50% neutralization titer (NT50) was calculated as the half-maximal inhibitory concentration values using four-parameter nonlinear regression in GraphPad Prism.

#### Binding antibody assay

High-binding 96-well flat bottom plates (Sarstedt AG & Co.KG, Nümbrecht, Germany) were coated with 1x10^8^ viral particles per well (using the same vectors as for the immunization of the mice from which the tested serum samples were obtained) in 50 μL coating buffer over night at 4 °C. Plates were washed twice with PBS-T (0.05% Tween-20/PBS), then blocked with 200 μL 5% BSA/PBS-T at room temperature for 45 min. A 2-fold serial dilution of mouse sera in blocking solution was performed and added to the plates after washing five times with PBS-T. Plates were incubated at 37 °C for 1 h and subsequently washed five times with PBS-T. Peroxidase-conjugated polyclonal anti-human antibody (1:2000 in blocking solution; Abcam, Cambridge, United Kingdom) was added and the plates were incubated at 37 °C for 70 min. After washing five times with PBS-T, colorimetric reaction was started by addition of 100 μL of a σ-phenylenediamine-dihydrochloride substrate solution (1 tablet in 0.05 M phosphate-citrate buffer; Merck KGaA, Darmstadt, Germany). The reaction was stopped with 1 M sulfuric acid and the absorbance at a wavelength of 491 nm was measured using the SPECTROstar nano (BMG LABTECH GmbH, Ortenberg, Germany). Arbitrary units (Figure 5H) are computed as ln(1000 x A_491nm_); the limit of detection corresponds to the maximum value reached by negative controls (background); and samples below this limit arbitrarily receive the value of 0 units.

### Quantification and statistical analyses

In ELISA-qPCR screens of vector collections (Figures 1C, 1D, and 1F), usual statistical tests were irrelevant given that our goal was to identify vectors which do not bind to PF4, not those that significantly bind to it. Therefore, non-binding vectors were considered to be those for which the number of bound VPs in PF4-coated wells overlapped with that in non-coated wells in all experiment repeats. This corresponds to a PF4 binding index consistently negative. The index was computed as follows for each experiment repeat from the qPCR-measured numbers of VPs bound in PF4-coated or uncoated wells (both conditions in triplicate):

$$\text{PF}4\;\text{binding}\;\text{index}=\left({\text{Minimum}}_{\text{PF}4}-{\text{Maximum}}_{\text{uncoated}}\right)\;/\;{\text{Average}}_{\text{uncoated}}$$

Measurements displayed in different subplot are taken from different samples. Error bars indicate the standard deviation. When N≥5, pairwise comparisons were performed using two-sided Mann-Whitney U tests when applicable. Multiple comparisons were conducted with two-way ANOVA or with one-way ANOVA and post-hoc Dunnett test comparisons with a control sample. The significance threshold was set at p < 0.05. Significance symbols: ns = non-significant, ∗ = p < 0.05, ∗∗ = p < 0.01, ∗∗∗ = p < 0.001. Statistical analyses were performed with the R software with the packages dplyr and ggplot2.
