# Supplementary material for: Novel adenovirus vaccine vectors lacking thrombosis-associated interactions with platelet factor 4
Source: iScience. 2025 Dec 4;29(1):114329. doi: 10.1016/j.isci.2025.114329 (PMC12775996; doi:10.1016/j.isci.2025.114329)
Supplement: Document S1. Figures S1–S6 and Tables S1 and S2 [file mmc1.pdf]

**iScience, Volume *29***

## Supplemental information

# Novel adenovirus vaccine vectors lacking thrombosis-associated interactions with platelet factor 4

**Erwan Sallard, Daniel Pembaur, Matias Ciancaglini, Lucie Manov-Bouard, Denice Weklak, Firas Hamdan, Chun Kit Chan, Franziska Jönsson, Elise Chabot, Carmen Musielak, Elena Scurti, Sara Feola, Sebastian Schellhorn, Nissai Beaude, Katrin Schröer, Daipayan Sarkar, Georgia Koukou, Xiaoyan Wang, Natascha Schmidt, Wibke Bayer, Malik Aydin, Vera Kemp, Alan L. Parker, Dirk Grimm, Tapani Viitala, Vincenzo Cerullo, Abhishek Singharoy, Alexander T. Baker, Wenli Zhang, Daniel Pinschewer, Florian Kreppel, and Anja Ehrhardt**

*Supplemental information titles and legends*

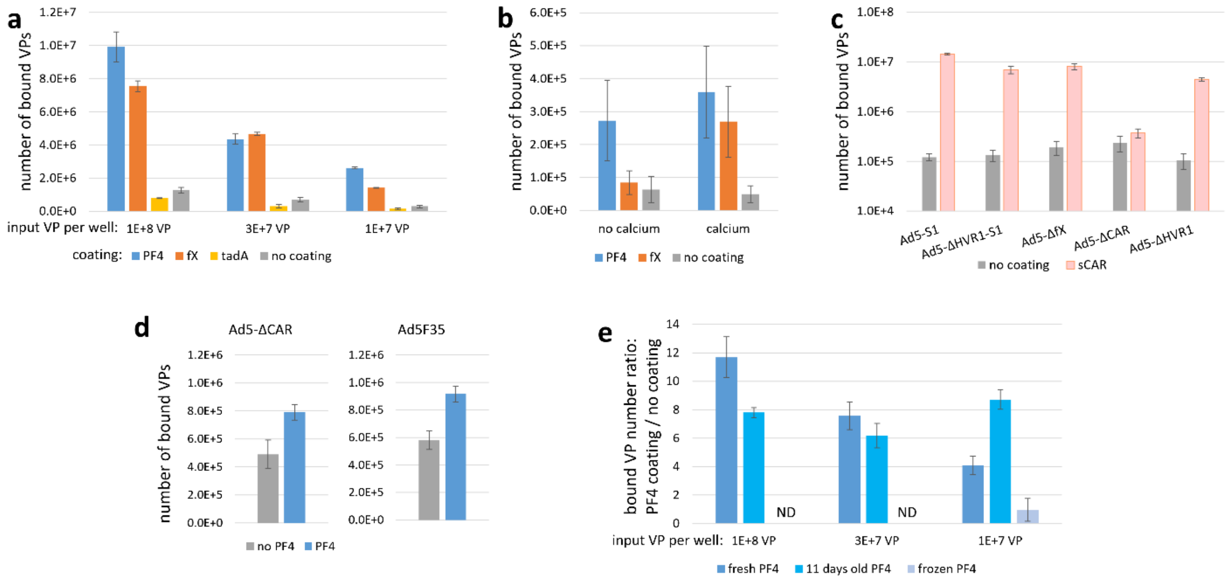


**Figure S1. Establishment of the ELISA-qPCR technique.**

**a:** The ELISA-qPCR technique facilitates specific and sensitive assessment of Ad5-protein interactions. ELISA plates were coated with PF4, factor X (fX), S.typhimurium tRNA-specific adenosine deaminase (tadA) or buffer only (no coating). Ad5-GLN was allowed to interact with coated proteins at doses of 1e8 (left), 3e7 (center) or 1e7 (right) virus particles (VP) per well, with the addition of 2.5mM CaCl2 in factor X samples. At all doses, the binding of Ad5-GLN to PF4 and fX (positive controls) was clearly detectable, while no increase in VP number in tadA-coated wells (negative controls) could be observed compared to uncoated wells. N=2 data points per sample. **b:** The ELISA-qPCR technique recapitulates the finding that Ad5 binding to factor X is calcium-dependent. Ad5-GLN was allowed to interact with PF4 or factor X (fX) in presence (“calcium”) or absence (“no calcium”) of a physiological concentration of 2.5mM CaCl2. N=6, two independent repeats. **c:** ELISA-qPCR recovery rate is consistent between Ad5 variants. Vector binding rates to wells coated with soluble CAR receptors (sCAR) or buffer only (no coating) were similar for all tested Ad5 variants that differed in non-structural genes or in their hexon protein (not involved in CAR binding), whereas the negative control Ad5-ΔCAR did not display significant binding to sCAR. N=6 data points per sample. **d:** Fiber-modified Ad5 variants bind PF4. The binding of Ad5F35 (Ad5 pseudotyped with Ad35 fiber) and Ad5-ΔCAR (carrying the fiber Y477A mutation ablating CAR binding) to PF4 was tested by ELISA-qPCR. Since our null hypothesis was that the bound VP numbers are significantly different (PF4 binding), statistical tests could not be applied. N=3. **e:** The PF4 protein remains stable when conserved at 4°C for several weeks but can not be used for ELISA-qPCR after freezing. The ratio of bound Ad5-GLN VP number between PF4-coated wells and non-coated wells was calculated after ELISA-qPCR. PF4 proteins were either used directly after resuspension of the lyophilised powder, after 11 days at 4°C or after freezing. N≥2 data points per sample. ND: no data.

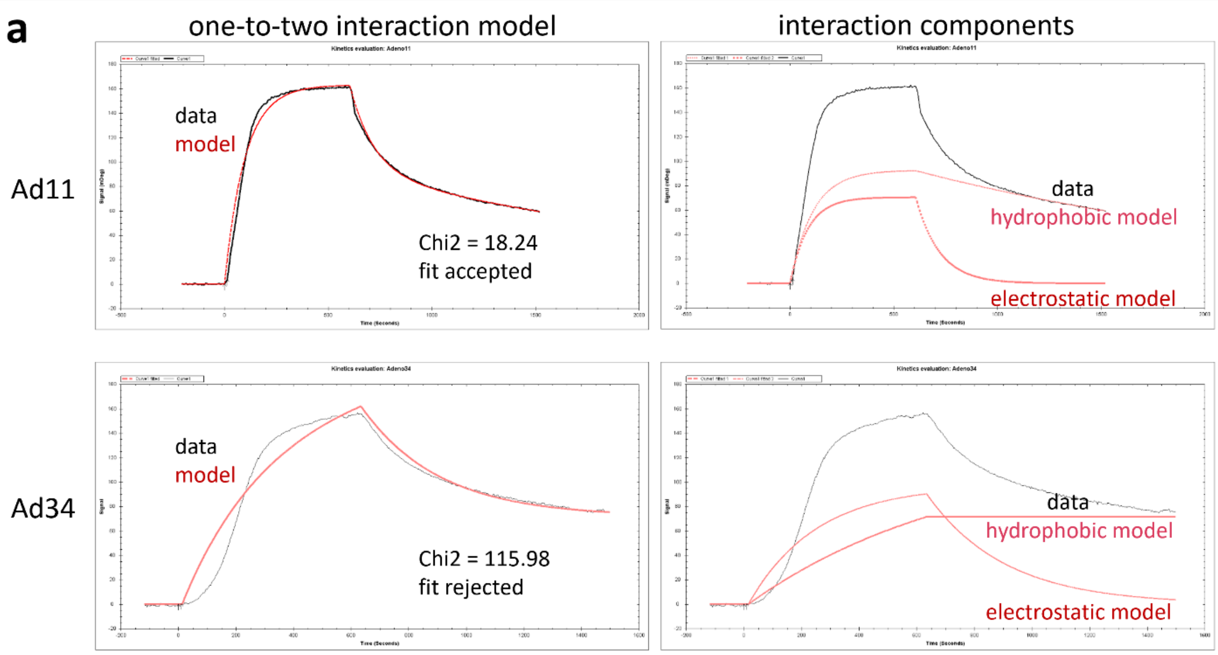


**b**

| | hydrophobic interaction | | | electrostatic interaction | | |
|---|---|---|---|---|---|---|
| | $k_{on}$ ($M^{-1}s^{-1}$) | $k_{off}$ ($s^{-1}$) | Kd (M) | $k_{on}$ ($M^{-1}s^{-1}$) | $k_{off}$ ($s^{-1}$) | Kd (M) |
| Ad5 | 3.40E4 | 8.30E-4 | 2.44E-8 | 1.18E4 | 1.24E-2 | 1.05E-6 |
| Ad11 | 3.59E4 | 4.82E-4 | 1.34E-8 | 1.18E4 | 8.79E-3 | 7.42E-7 |
| Ad13 | 2.83E4 | 3.63E-4 | 1.29E-8 | 9.83E3 | 7.61E-3 | 7.74E-7 |

**Figure S2. Ad-PF4 interactions measured by surface plasmon resonance fit a one-to-two interaction model.**

**a:** A one-to-two interaction model consisting of a fast and a slow component ("electrostatic model" and "hydrophobic model" respectively) was found to be the best fitting to experimental curve (data) for Ad5, Ad11 and Ad13, as shown here with Ad11. This model was however poorly fitting to Ad34 (displayed here), Ad80 and HVR1-deleted Ad5. Please note that the number of model interaction components does not indicate the valency of either interaction partner. **b:** Kinetic parameters of the one-to-two interaction model were calculated for both of its components for the Ad types for which the model was not rejected.

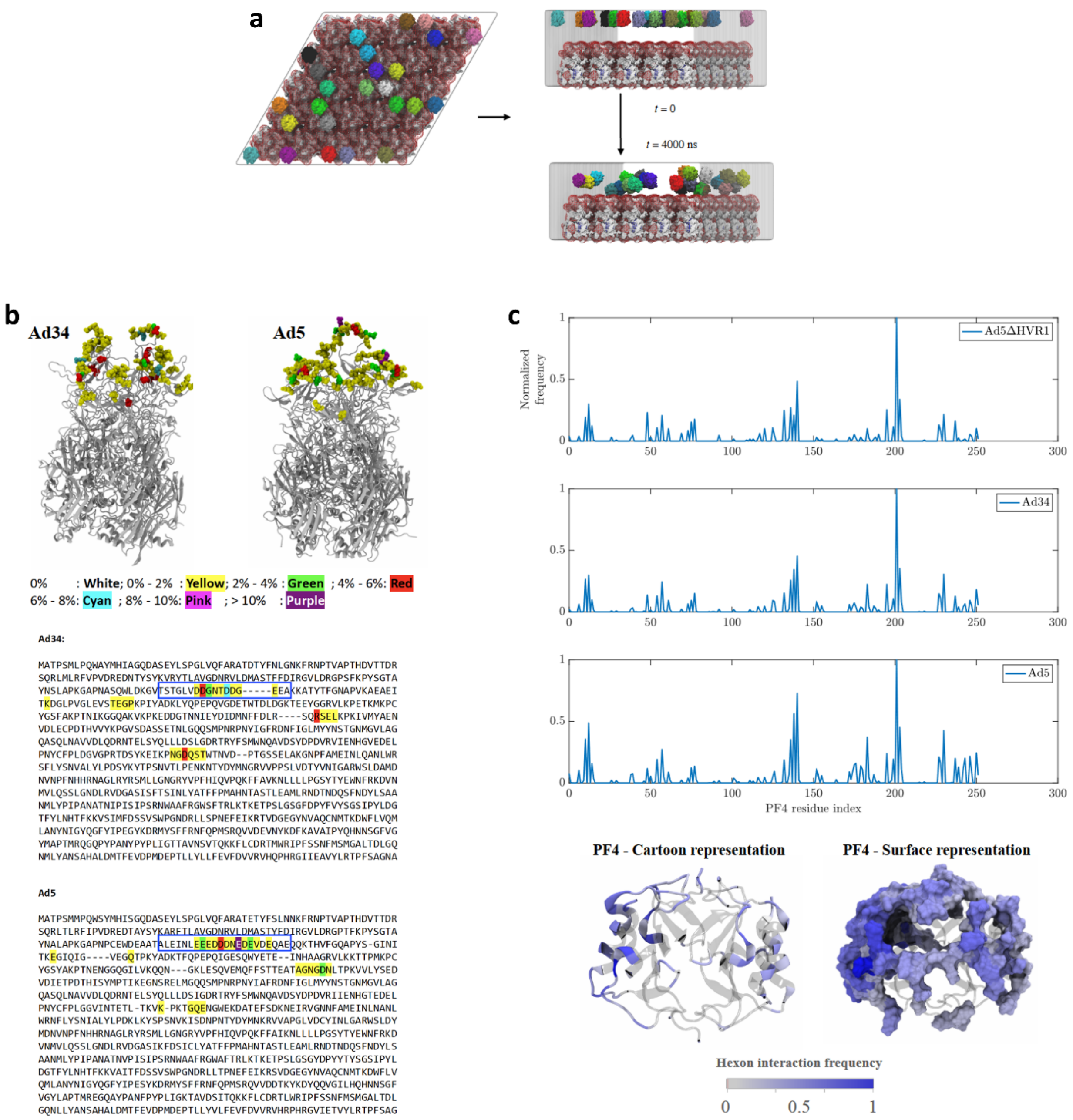


**Figure S3. Structural modeling of PF4-hexon interactions.**

**a**: Simulation setup for Brownian Dynamics (BD) simulations. First (left), 16 replicas of simulations were generated. Each replica consists of 25 copies of PF4 tetramers (giving 400 copies in total) randomly placed 150 Å over the hexon sheets at initializations. Then (right), each of these replicas was simulated for 4 microseconds to sample PF4s-hexon interaction. The diffusion boundary, which is shown as the grey box above, is directly derived from the periodic boundary from molecular dynamics (MD) simulations on the hexon sheet to facilitate future MD refinement or free energy calculations**. b:** BD modeling of hexon residues probability of interaction with PF4. For both Ad34's hexon and Ad5's hexon, residues that interact with PF4 are shown by bead representations of their atoms with colors indicating the frequency of PF4 occupancy sampled through BD simulations. These PF4-interacting residues are also highlighted with respect to their positions in the respective sequence of Ad34's hexon and that of Ad5's hexon. Regions corresponding to HVR1s are highlighted by blue boxes. **c:** For each PF4 residue in a tetramer, its frequency to interact with hexons of either Ad34, Ad5, or

Ad5ΔHVR1 was sampled from BD simulations, and was normalized to the maximal value observed in each individual case. The resulting profiles were highly similar for all 3 adenoviruses, namely Ad34, Ad5, and Ad5ΔHVR1. Below, each residue is colored according to its hexon interaction frequency, as being averaged over the 3 cases of Ad34, Ad5, and Ad5ΔHVR1. The six N-terminal residues of each PF4 monomer are absent from the structures and the residues indexing.

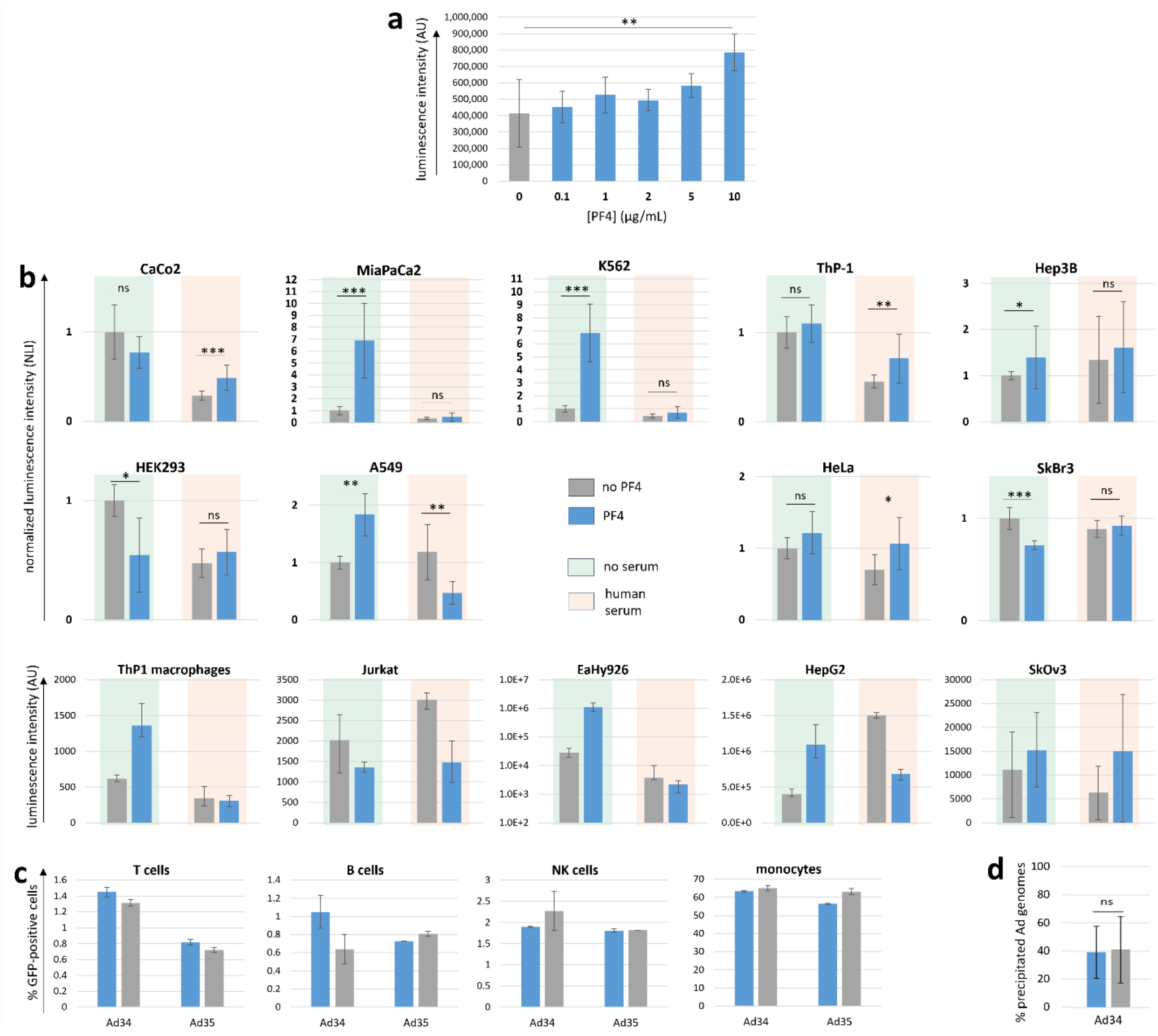


**Figure S4. Infectivity assays on an array of immortalized and primary cell lines**.

**a:** Ad5 vectors from the Ad-GLN collection were incubated with varying concentrations of PF4 then allowed to infect A549 cells at 500 vpc. Ad-expressed luciferase luminescence was quantified 24 hpi. N≥4. A Welch ANOVA test indicated an influence of PF4 concentration on infectivity levels ($p = 0.0035$) and post-hoc Dunnett T3 tests were performed to compare each sample with the "no PF4" control; only significant p-values of the post-hoc test are displayed. **b, c:** Infectivity assays were conducted as in Fig. 4. **b:** Immortalized cells were infected with 20 vpc of Ad5 vector from the Ad-GLN collection. Ad-expressed luciferase luminescence was quantified 24 hpi and normalized on the average of the "no PF4, no serum" condition for each cell line. N≥4, one to three independent repeats. **c:** Primary peripheral blood mononuclear cells were infected with 2000 vpc of Ad34 or Ad35 vectors from the Ad-GLN collection. Ad-expressed GFP fluorescence was quantified 48 hpi and the proportions of GFP-positive cells were normalized on the average of the "no PF4, FBS" condition for each cell type. N=2. **d:** Erythrocyte pull-down of Ad34 from the Ad-GLN collection in absence or presence of PF4. N=10, three independent repeats. A pairwise comparison was conducted with the Mann-Whitney U test. Error bars indicate the standard deviation. The significance threshold was set at $p<0.05$. Significance symbols: ns = non-significant, * = $p<0.05$, ** = $p<0.01$, *** = $p<0.001$.

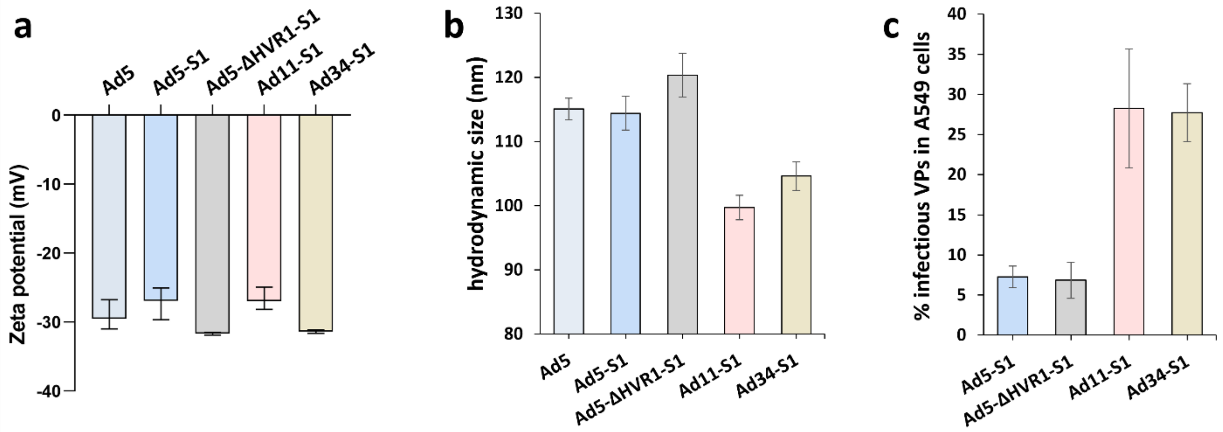


**Figure S5. Vaccine vectors quality controls.**

**a:** Surface potential of Ad particles measured by electrophoretic light scattering (ELS). N=3. **b:** Hydrodynamic diameter of Ad particles measured by ELS. N=6. **c:** Vectors were allowed to infect A549 cells at 20vpc and internalized Ad genomes were titrated by qPCR at 3 hpi to calculate the proportion of infectious particles in the preparations. These percentages may not be representative of infectivity levels in mouse tissues. N=4. Error bars indicate the standard deviation.

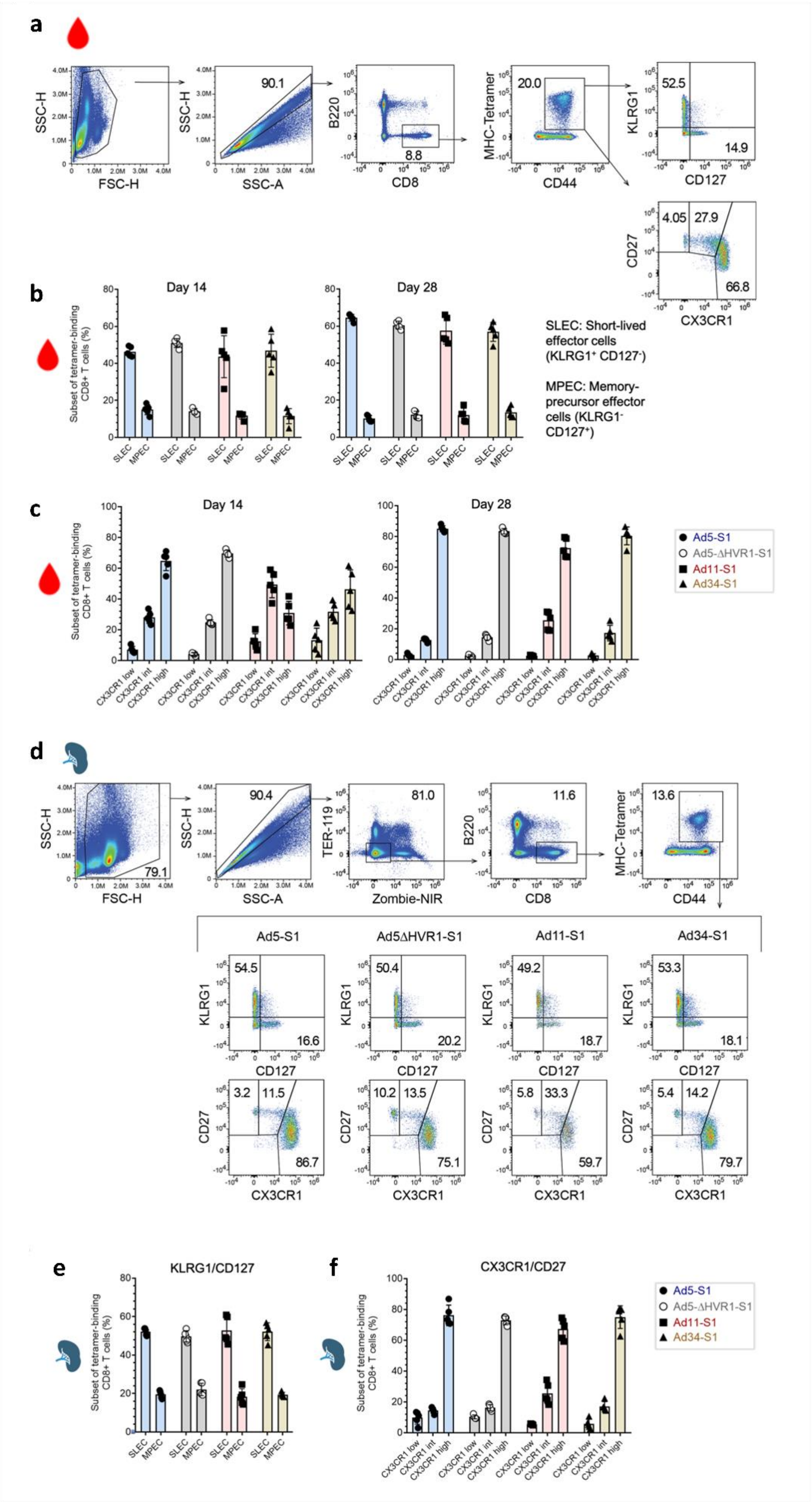


**Figure S6. Phenotyping of S1-specific T cells from immunized mice.**

**a**: Gating strategy to determine the frequencies and phenotypes of S1-epitope specific CD8+ T cells in peripheral blood. **b, c:** Frequencies of S1-specific CD8+ T cells based on KLRG1 and CD127 **(b)** or CX3CR1 and CD27 **(c)** at day 14 or 28 after immunization. **d:** Gating strategy to

determine the frequencies and phenotypes of S1-epitope specific CD8+ T cells in spleens. Representative FACS plots of each group phenotype are shown. **e, f:** Frequencies of S1-specific CD8+ T cells based on KLRG1 and CD127 **(e)** or CX3CR1 and CD27 **(f)** at day 30 after immunization. Error bars indicate the standard deviation.

**Table S1. Sequence of the mouse PF4 expression plasmid.**
Mouse PF4 coding sequence is represented in uppercase.

gacggatcgggagatctcccgatcccctatggtgcactctcagtacaatctgctctgatgccgcatagttaagccagtatctgctccctg
cttgtgtgttggaggtcgctgagtagtgcgcgagcaaaatttaagctacaacaaggcaaggcttgaccgacaattgcatgaagaatct
gcttagggttaggcgttttgcgctgcttcgcgatgtacgggccagatatacgcgttgacattgattattgactagttattaatagtaatca
attacggggtcattagttcatagcccatatatggagttccgcgttacataacttacggtaaatggcccgcctggctgaccgcccaacga
cccccgcccattgacgtcaataatgacgtatgttcccatagtaacgccaatagggactttccattgacgtcaatgggtggagtatttacg
gtaaactgcccacttggcagtacatcaagtgtatcatatgccaagtacgccccctattgacgtcaatgacggtaaatggcccgcctggc
attatgcccagtacatgaccttatgggactttcctacttggcagtacatctacgtattagtcatcgctattaccatggtgatgcggttttgg
cagtacatcaatgggcgtggatagcggtttgactcacggggatttccaagtctccaccccattgacgtcaatgggagtttgttttggcac
caaaatcaacgggactttccaaaatgtcgtaacaactccgccccattgacgcaaatgggcggtaggcgtgtacggtgggaggtctat
ataagcagagctctctggctaactagagaacccactgcttactggcttatcgaaattaatacgactcactatagggagacccaagctg
ATGAGCGTCGCTGCGGTGTTTCGAGGCCTCCGGCCCAGTCCTGAGCTGCTGCTTCTGGGCCTGTTGTT
TCTGCCAGCGGTGGTTGCTGTCACCAGCGCTGGTCCCGAAGAAAGCGATGGAGATCTTAGCTGTGTGT
GTGTGAAGACCATCTCCTCTGGGATCCATCTTAAGCACATCACCAGCCTGGAGGTGATCAAGGCAGGA
CGCCACTGTGCGGTTCCCCAGCTCATAGCCACCCTGAAGAATGGGAGGAAAATTTGCCTGGACCGGCA
AGCACCCCTATATAAGAAAGTAATCAAGAAAATCCTGGAGAGTtgataaacccgctgatcagcctcgactgtgcctt
ctagttgccagccatctgttgtttgcccctcccccgtgccttccttgaccctggaaggtgccactcccactgtcctttcctaataaaatgag
gaaattgcatcgcattgtctgagtaggtgtcattctattctggggggtggggtggggcaggacagcaagggggaggattgggaagac
aatagcaggcatgctggggatgcggtgggctctatggcttctgaggcggaaagaaccagctggggctctagggggtatccccacgcg
ccctgtagcggcgcattaagcgcggcgggtgtggtggttacgcgcagcgtgaccgctacacttgccagcgccctagcgcccgctccttt
cgctttcttcccttcctttctcgccacgttcgccggctttccccgtcaagctctaaatcgggggctccctttagggttccgatttagtgcttt
acggcacctcgaccccaaaaaacttgattagggtgatggttcacgtagtgggccatcgccctgatagacggtttttcgccctttgacgtt
ggagtccacgttctttaatagtggactcttgttccaaactggaacaacactcaaccctatctcggtctattcttttgatttataagggattt
tgccgatttcggcctattggttaaaaaatgagctgatttaacaaaaatttaacgcgaattaattctgtggaatgtgtgtcagttagggtgt
ggaaagtccccaggctccccagcaggcagaagtatgcaaagcatgcatctcaattagtcagcaaccaggtgtggaaagtccccagg
ctccccagcaggcagaagtatgcaaagcatgcatctcaattagtcagcaaccatagtcccgcccctaactccgcccatcccgccccta
actccgcccagttccgcccattctccgccccatggctgactaattttttttatttatgcagaggccgaggccgcctctgcctctgagctatt
ccagaagtagtgaggaggcttttttggaggcctaggcttttgcaaaaagctcccgggagcttgtatatccattttcggatctgatcaaga
gacaggatgaggatcgtttcgcatgattgaacaagatggattgcacgcaggttctccggccgcttgggtggagaggctattcggctatg
actgggcacaacagacaatcggctgctctgatgccgccgtgttccggctgtcagcgcaggggcgcccggttctttttgtcaagaccgac
ctgtccggtgccctgaatgaactgcaggacgaggcagcgcggctatcgtggctggccacgacgggcgttccttgcgcagctgtgctcg
acgttgtcactgaagcgggaagggactggctgctattgggcgaagtgccggggcaggatctcctgtcatctcaccttgctcctgccga
gaaagtatccatcatggctgatgcaatgcggcggctgcatacgcttgatccggctacctgcccattcgaccaccaagcgaaacatcgc
atcgagcgagcacgtactcggatggaagccggtcttgtcgatcaggatgatctggacgaagagcatcaggggctcgcgccagccgaa
ctgttcgccaggctcaaggcgcgcatgcccgacggcgaggatctcgtcgtgacccatggcgatgcctgcttgccgaatatcatggtgg
aaaatggccgcttttctggattcatcgactgtggccggctgggtgtggcggaccgctatcaggacatagcgttggctacccgtgatatt
gctgaagagcttggcggcgaatgggctgaccgcttcctcgtgctttacggtatcgccgctcccgattcgcagcgcatcgccttctatcgc
cttcttgacgagttcttctgagcgggactctggggttcgaaatgaccgaccaagcgacgcccaacctgccatcacgagatttcgattcc
accgccgccttctatgaaaggttgggcttcggaatcgttttccgggacgccggctggatgatcctccagcgcggggatctcatgctgga
gttcttcgcccaccccaacttgtttattgcagcttataatggttacaaataaagcaatagcatcacaaatttcacaaataaagcattttttt
cactgcattctagttgtggtttgtccaaactcatcaatgtatcttatcatgtctgtataccgtcgacctctagctagagcttggcgtaatcat
ggtcatagctgtttcctgtgtgaaattgttatccgctcacaattccacacaacatacgagccggaagcataaagtgtaaagcctggggt
gcctaatgagtgagctaactcacattaattgcgttgcgctcactgcccgctttccagtcgggaaacctgtcgtgccagctgcattaatga
atcggccaacgcgcggggagaggcggtttgcgtattgggcgctcttccgcttcctcgctcactgactcgctgcgctcggtcgttcggctg
cggcgagcggtatcagctcactcaaaggcggtaatacggttatccacagaatcaggggataacgcaggaaagaacatgtgagcaaa
aggccagcaaaaggccaggaaccgtaaaaaggccgcgttgctggcgtttttccataggctccgcccccctgacgagcatcacaaaaa

tcgacgctcaagtcagaggtggcgaaacccgacaggactataaagataccaggcgtttccccctggaagctccctcgtgcgctctcct
gttccgaccctgccgcttaccggatacctgtccgcctttctcccttcgggaagcgtggcgctttctcatagctcacgctgtaggtatctca
gttcggtgtaggtcgttcgctccaagctgggctgtgtgcacgaaccccccgttcagcccgaccgctgcgccttatccggtaactatcgtc
ttgagtccaacccggtaagacacgacttatcgccactggcagcagccactggtaacaggattagcagagcgaggtatgtaggcggtg
ctacagagttcttgaagtggtggcctaactacggctacactagaagaacagtatttggtatctgcgctctgctgaagccagttaccttcg
gaaaaagagttggtagctcttgatccggcaaacaaaccaccgctggtagcggtggtttttttgtttgcaagcagcagattacgcgcaga
aaaaaaggatctcaagaagatcctttgatcttttctacggggtctgacgctcagtggaacgaaaactcacgttaagggattttggtcat
gagattatcaaaaaggatcttcacctagatccttttaaattaaaaatgaagttttaaatcaatctaaagtatatatgagtaaacttggtct
gacagttaccaatgcttaatcagtgaggcacctatctcagcgatctgtctatttcgttcatccatagttgcctgactccccgtcgtgtagat
aactacgatacgggagggcttaccatctggccccagtgctgcaatgataccgcgagacccacgctcaccggctccagatttatcagca
ataaaccagccagccggaagggccgagcgcagaagtggtcctgcaactttatccgcctccatccagtctattaattgttgccgggaag
ctagagtaagtagttcgccagttaatagtttgcgcaacgttgttgccattgctacaggcatcgtggtgtcacgctcgtcgtttggtatggc
ttcattcagctccggttcccaacgatcaaggcgagttacatgatcccccatgttgtgcaaaaaagcggttagctccttcggtcctccgat
cgttgtcagaagtaagttggccgcagtgttatcactcatggttatggcagcactgcataattctcttactgtcatgccatccgtaagatgc
ttttctgtgactggtgagtactcaaccaagtcattctgagaatagtgtatgcggcgaccgagttgctcttgcccggcgtcaatacgggat
aataccgcgccacatagcagaactttaaaagtgctcatcattggaaaacgttcttcggggcgaaaactctcaaggatcttaccgctgtt
gagatccagttcgatgtaacccactcgtgcacccaactgatcttcagcatcttttactttcaccagcgtttctgggtgagcaaaaacagg
aaggcaaaatgccgcaaaaaagggaataagggcgacacggaaatgttgaatactcatactcttcctttttcaatattattgaagcattt
atcagggttattgtctcatgagcggatacatatttgaatgtatttagaaaaataaacaaataggggttccgcgcacatttccccgaaaa
gtgccacctgacgtc

| name | sequence | target |
|---|---|---|
| GLN-for | accaagcgaaacatcgcatcgag | vectors of the Ad-GLN collection |
| GLN-rev | gcgataccgtaaagcacgaggaag | vectors of the Ad-GLN collection |
| WT-for | GCCCCAGTGGTCTTACATGCACATC | vectors of the Ad-WT collection |
| WT-rev | GCCACGGTGGGGTTTCTAAACTT | vectors of the Ad-WT collection |
| WT-probe | CCGGGTCTGGTGCAGTTTGCCCGC | vectors of the Ad-WT collection |
| CMV-for | tacatcaatgggcgtggata | vaccine-equivalent vectors |
| CMV-rev | ggcggagttgttacgacatt | vaccine-equivalent vectors |
| Fiber-for | accggtttccgtgtcatatgg | Ad5 hexon mutants and Ad5-ΔCAR |
| Fiber-rev | ggtattgcagcttcctcctgg | Ad5 hexon mutants and Ad5-ΔCAR |
| AAV-for | aacgccaatagggactttcc | vectors of the AAV collection |
| AAV-rev | gggcgtacttggcatatgat | vectors of the AAV collection |
| cell-for | GGAATTGATTTGGGAGAGCATC | human beta-2-microglobulin |
| cell-rev | CAGGTCCTGGCTCTACAATTTACTA | human beta-2-microglobulin |
| cell-probe | GAAGGTGGATGATCTGCCCAGTCACACT | human beta-2-microglobulin |

**Table S2. Primers used in this study.**
